# A Single‐Cell Transcriptome Atlas Characterizes the Immune Landscape of Human Testes During Aging

**DOI:** 10.1111/acel.70032

**Published:** 2025-03-06

**Authors:** Qiaoling Jiang, Lina Cui, Xichen Nie, Hui Cai, Wenxiu Zhang, Xiaojian Lu, Yifei Guo, James M. Hotaling, Bradley R. Cairns, Xiaoyan Wang, Jingtao Guo

**Affiliations:** ^1^ The First Affiliated Hospital of USTC, Division of Life Sciences and Medicine University of Science and Technology of China Hefei China; ^2^ State Key Laboratory of Organ Regeneration and Reconstruction Institute of Zoology, Chinese Academy of Sciences Beijing China; ^3^ Beijing Institute for Stem Cell and Regenerative Medicine Beijing China; ^4^ University of Chinese Academy of Sciences Beijing China; ^5^ Howard Hughes Medical Institute, Department of Oncological Sciences and Huntsman Cancer Institute Spencer Fox Eccles School of Medicine, University of Utah Salt Lake City Utah USA; ^6^ Division of Urology, Department of Surgery Spencer Fox Eccles School of Medicine, University of Utah Salt Lake City Utah USA

**Keywords:** aging, human testis, immune cells, single‐cell RNA‐seq

## Abstract

Aging disrupts immune regulation, affecting tissue function and increasing vulnerability to various diseases. However, the effects of aging on immune cells within human testes are not well understood. In this study, we utilized single‐cell RNA sequencing to profile immune cells from 33 human testis samples from individuals aged 21 to 69. Our analysis revealed key immune cell types, including CD8^+^ T cells, monocytes, cDC2 cells, and various macrophage subtypes within the testes. We observed an age‐related change in monocytes and MRC1^hi^ tissue‐resident macrophage (TRM), a pattern consistent in both human and mouse testes. Individuals aged 40 and older showed notable shifts in pathways related to phagocytosis, cytokine signaling, and antigen presentation. Monocytes also exhibited pro‐inflammatory characteristics, potentially contributing to the low‐grade inflammation commonly associated with aging. Together, these findings provide insights into age‐related immune cell alterations in human testes and uncover molecular mechanisms underlying these shifts, offering a valuable resource for understanding immune aging in the reproductive system.

## Graphical abstract

This study utilized single‐cell RNA sequencing to analyze immune cells in 33 human testis samples (ages 21–69). We found age‐related alterations in monocytes and MRC1^hi^ TRM, with shifts in phagocytosis, cytokine, and antigen presentation, contributing to low‐grade inflammation in aging. These findings provide insights into immune aging in human testis.

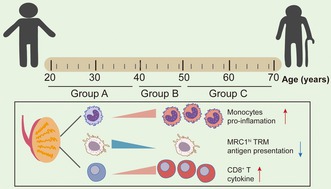

## 1 Introduction

The aging process disrupts immune regulation, which concurrently impacts tissue functions and increases susceptibility to various diseases (Franceschi et al. 2018; Hou et al. 2024; Mogilenko et al. 2022; Terekhova et al. 2023; Yousefzadeh et al. 2021). Fully understanding the age‐related immune process requires a multifaceted perspective, as immune cell composition is shaped by anatomical location, general health, and biological age (Ross et al. 2024; Wang et al. 2020; Yousefzadeh et al. 2021). Indeed, extensive studies have documented age‐related changes in immune cell composition in peripheral blood mononuclear cells (PBMC) Wang et al. 2025 and organs such as the lungs, spleen, kidney, and ovary (De Silva et al. 2023; Palacio et al. 2019; Sen et al. 2020; Winkler et al. 2024). In PBMC, immune homeostasis shifts with age, characterized by changes in multiple subpopulations including the accumulation of GZMK^+^CD8^+^ T cells and HLA‐DR^+^CD4^+^ T cells (Terekhova et al. 2023). In organs, immune cell alterations often lead to dysfunction (Yousefzadeh et al. 2021). In the ovary, for example, age‐related enrichment of pyroptotic monocyte‐derived macrophages fosters a pro‐inflammatory environment, accelerating stromal cell senescence and impacting fertility (Zhou et al. 2024).

Despite its critical role in male fertility, limited research has explored how age‐related changes in the immune microenvironment impact testicular cells. Studies on older men indicate a decline in gonadal function and fertility with age (Cui et al. 2025; Nie et al. 2022; Zhuang et al. 2023). This physiological shift may be associated with various alterations within the testis, particularly in the function and composition of immune cells. These immune changes may not only affect fertility but also have broader implications for systemic health, as an aged immune system plays a causal role in overall aging (Yousefzadeh et al. 2021). Recent studies highlight that immune cells play a substantial role in the testes (Barrachina et al. 2023; Hasan et al. 2024; Wang, Liu, et al. 2024; Yang et al. 2024), with age‐related shifts in testicular immune cells documented in both mice and humans (Endo et al. 2024; Krishnarajah et al. 2022; Nie et al. 2022; Zhang et al. 2023). Aged testes exhibit heightened inflammatory responses, increased eosinophil chemotaxis, and elevated levels of chemokine CCL8 (Endo et al. 2024; Han et al. 2021). Age‐related changes in macrophage subpopulations, antigen presentation, and complement pathways have also been noted, with additional findings in aged mouse testes showing immune‐related changes such as regulation of endocytosis and mononuclear cell proliferation (Zhang et al. 2023).

Similarly, aging human testes display alterations in macrophage‐related pathways, including the MIF signaling pathway and the complement system, as well as upregulation in inflammatory genes such as *FCER1A*, *CLEC10A*, *CXCR4*, and *MIF*, associated with immune activation (He et al. 2021; Korbecki et al. 2024). Moreover, the cross talk between macrophages and somatic cells has changed, indicating that macrophages potentially coordinate with other cell types to influence testis aging (Nie et al. 2022). However, due to limitations in study methods and sample sizes, previous researches have largely focused on macrophages, leaving other immune cell types, such as T cells and monocytes, less examined despite their key roles in aging tissue (Carrasco et al. 2022; Chambers et al. 2021). Furthermore, prior studies have typically focused on only two age groups—young and old—obscuring the more gradual immune changes that occur during aging. It remains unclear whether these changes manifest only in advanced age or if they begin earlier and progress over time, limiting our understanding of the nuanced immune cell dynamics in testis aging.

To address these gaps, we analyzed single‐cell transcriptomic data from the testes of 33 adult individuals aged 21 to 69. We identified previously uncharacterized immune cell subtypes in the human testes, including monocytes, cDC2 cells, and multiple macrophage subtypes, thereby revealing specific age‐related changes within these cells. Our findings showed that significant immune alterations begin in individuals in their 40s and progressively increase with age. Among lymphocytes, CD8^+^ T cells demonstrated heightened activation and increased MHC class I expression. In the myeloid lineage, monocytes infiltration increased with age, potentially differentiating into other macrophage subtypes and contributing to low‐grade inflammation over time. This early immune activation, observable in individuals in their 40s, suggests that age‐related changes in the testicular immune microenvironment may start earlier and accumulate gradually. Collectively, these findings indicated that age‐associated changes in human testicular immune cells emerge before typical physiological aging, mapping critical molecular and cellular transitions in testicular immune aging and offering a valuable resource for understanding human aging.

## 2 Results

### 2.1 Single‐Cell Transcriptome Analysis of Immune Cells in Human Testes During Aging

**FIGURE 1:**
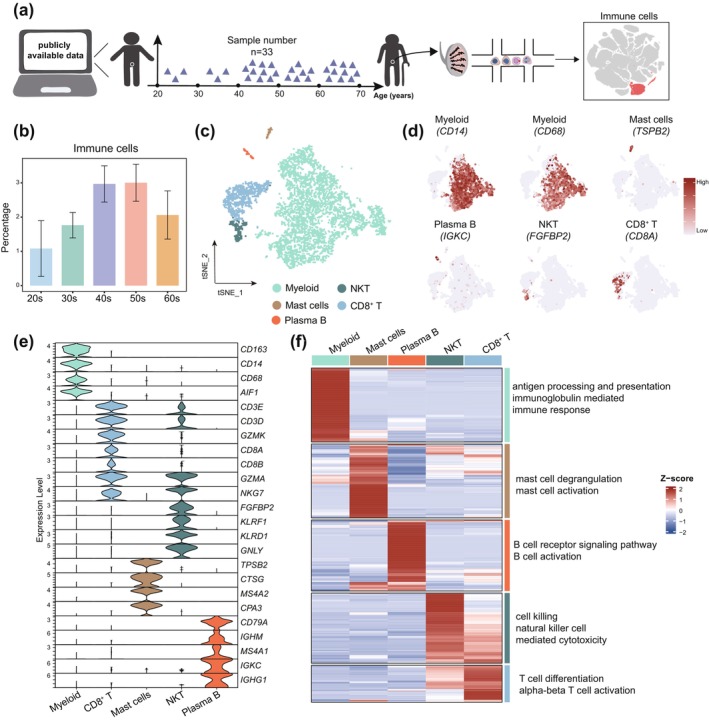
Single‐cell analysis of immune cells in human testis during aging. (a) Schematic illustration of the workflow includes sample information, categorized by age groups: 20–30 years (*n* = 3), 30–40 years (*n* = 3), 40–50 years (*n* = 10), 50–60 years (*n* = 7), 60–70 years (*n* = 10). (b) Bar plot showing the percentage of immune cells in human testis for each group, and colors represent different groups. Bars represent the mean with SD. (c) t‐Distributed Stochastic Neighbor Embedding (t‐SNE) plot showing the major immune cell types (*n* = 5299 cells) from samples. Dots represent individual cells, and colors represent different cell types. (d) Expression of selected markers identifying major immune cell types cast on the t‐SNE plot. Red (or gray) represents a high (or low) expression level as shown on the color key at the right. (e) Violin plots showing the expression of more selected markers identifying major immune cell types, and colors represent different cell types. (f) Left: Heatmap showing the top 100 differentially expressed genes of each cell cluster from (c). The scaled gene expression levels are colored according to *Z* score. Right: The corresponding top two GO terms enriched in the marker genes of each cell cluster, with *Z* score colored according to the color key at the right.

To explore age‐associated immune cell changes within the human testis, we conducted a comprehensive reanalysis of a single‐cell transcriptomic dataset from 33 males aged 21 to 69 years (Figure 1a). To capture the nuanced changes occurring at different stages of life, we divided the samples into five age splits, each spanning a decade: 20s, 30s, 40s, 50s, and 60s. This stratification allowed us to track immune cell shifts more precisely over time and pinpoint when significant age‐related changes in the testicular immune microenvironment begin.

To evaluate potential batch effects, we performed correlation analysis on samples and found that most exhibited a high correlation coefficient, suggesting minimal batch effects (Figure S1a). Following rigorous quality control, we identified the major cell types present in the testes, basing classifications on well‐defined marker genes for each cell type (Figure S1b,c,e). Sertoli cells decreased during aging, consistent with previous studies (Huang et al. 2023; Nie et al. 2022) (Figure S1d). Notably, we observed a substantial increase in the percentage of immune cells beginning in the 40s, a finding that suggests a marked shift in the immune landscape of the testis associated with aging (Figure 1b). This trend indicates that the 40s may represent a potential acceleration in immune cell involvement and testicular reorganization, with a stable pattern observed in the subsequent older age groups.

Focusing specifically on immune cells, we conducted a subcluster analysis, isolating 6017 immune cells for further examination. Utilizing 2000 highly variable genes, we performed PCA and t‐SNE analysis following data normalization and scaling with Seurat. As a result, we identified five major clusters, including myeloid cells (*CD68* ^+^, *CD14* ^+^, *CD163* ^+^), mast cells (*CPA3* ^+^, *MS4A2* ^+^, *CSTG* ^+^), plasma B cells (*IGHG1* ^+^, *IGKC* ^+^, *CD79A* ^+^), natural killer T cells (NKT; *KLRF1* ^+^, *FGFBP2* ^+^, *CD3D* ^+^), and CD8^+^ T cells (*CD8B* ^+^, *CD8A* ^+^, *GZMK* ^+^) (Figure 1c–e). We clustered the marker genes specific to each cell type and annotated them with Gene Ontology (GO) terms (Figure 1f).

### 2.2 Increase in Age‐Related Features Among Immune Cells in 40s

**FIGURE 2:**
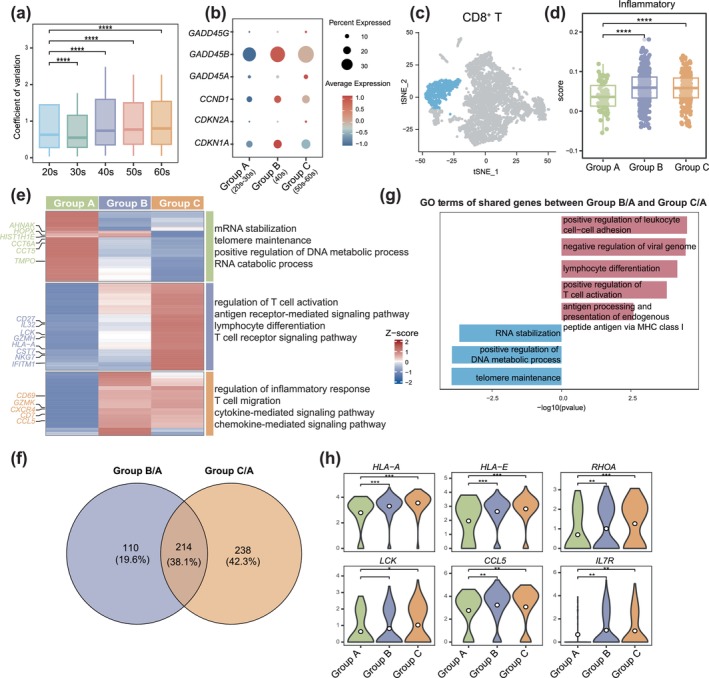
Age‐related features increased in Group B. (a) Box plot showing the value of the coefficient of variation (c.v.) of immune cells for each group. The c.v. is significantly changed in the 40s. Box indicates the range from the 25th to the 75th percentile; center lines indicate the median. The *p* value was calculated by Student's *t*‐test. ****p < 0.0001. (b) Dot plots showing the average expression of aging marker genes among three age groups. Color represents the average expression level. (c) t‐SNE plot showing the projection of CD8^+^ T cells from (Figure 1c). (d) The signature score of inflammatory response in CD8^+^ T cells among three age groups. Color indicates the age groups, box indicates range from the 25th to 75th percentile; center lines indicate median, dot represents individual cells. The *p* value was calculated by Student's *t*‐test. ****p < 0.0001. (e) Left: Heatmap showing the differentially expressed genes of each age group in CD8^+^ T cells. Right: The corresponding top four GO terms enriched in these genes. The scaled gene expression levels are colored according to *Z*‐score. (f) Venn diagram showing the number of significantly DEGs in CD8^+^ T cells and their overlap. (g) Representative GO terms enrichment of overlap genes from (f) and their associated *p* value. (h) Violin plots showing the expression levels of some shared and upregulated genes in different age groups. The white dot inside the violin represents the mean. Wilcox test,   ***p ≤ 0.001; **p ≤ 0.01; *p ≤ 0.05.

To closely assess immune cell dynamics with age, we conducted a coefficient of variation analysis on immune cells and found a significant increase in gene expression variability beginning in the 40s (Figure 2a) and (Figure S2a). This suggests that immune cells become notably more susceptible to age‐related changes in this decade, a period recognized as critical in the aging process due to marked shifts in molecular markers and physiological functions that heighten vulnerability to age‐related diseases (Shen et al. 2024). Additionally, prior studies have documented substantial declines in both T cell diversity and immune function in individuals during their 40s (Simnica et al. 2019), further indicating that immune stability may be particularly affected during this period.

Our findings, along with prior studies, suggest that the 40s represent a phase of pronounced change for testicular immune cells. To better explore these changes, we classified the data into three age groups: individuals younger than 40 years as Group A, those aged 40–50 years as Group B, and those aged 50–69 years as Group C. Upon comparing immune cell counts across these groups, we observed an increase in CD8^+^ T cells in Group B, alongside a decrease in myeloid cells—trends that continued into Group C (Figure S2b).

We also evaluated the expression of key aging markers across the three age groups, noting that genes such as *CDKN1A* and *CDKN2A* (Hou et al. 2024; Suryadevara et al. 2024), along with age‐associated genes *GADD45A* and *GADD45B* (Diao et al. 2018; Zhan et al. 2024), showed significantly higher expression levels in both Groups B and C (Figure 2b). These patterns suggest that age‐related immune changes begin manifesting notably in Group B, underscoring the 40s as a critical period for the onset of immune aging in the testes.

### 2.3 Increased CD8 ^+^ T‐Cell Activation in Testes From Group B and Group C

CD8^+^ T cells are crucial for mediating immune responses and regulating inflammation (Groh et al. 2021; Kaya et al. 2022). To investigate the age‐related alterations of CD8^+^ T cells, we compared the cell number among the three age groups and observed a significant increase starting in Group B (Figure S2b,c). The inflammatory response‐signaling pathway is the key mechanism through which immune cells activate immune responses (Ren et al. 2021; Zhu et al. 2023). We quantitatively assessed the inflammatory state of CD8^+^ T cells (Figure 2c) by calculating inflammatory scores based on the expression of the inflammatory response gene set. Group B exhibited a substantial increase in inflammatory scores, while Group C maintained relatively stable scores (Figure 2d). This suggests a marked enhancement of CD8^+^ T cell immune responses during aging.

To further explore the molecular mechanisms affecting CD8^+^ T cells with age, we conducted differential gene expression (DEG) analysis across the three groups. Notably, CD8^+^ T cells from Group A exhibited a significant enrichment of highly expressed genes related to telomere maintenance and mRNA stabilization compared to Groups B and C. The expression levels of these genes demonstrated a marked decline in Groups B and C, reflecting classic age‐related changes (Blackburn et al. 2015; Li et al. 2023). In contrast, genes that were highly expressed in Groups B and C were enriched in T cell activation and cytokine‐mediated signaling pathways, indicating an enhanced immune response that may contribute to the low‐grade inflammation commonly associated with aging (Figure 2e). In Group A, we observed higher expression levels of *TMPO* and *AHNAK*, both of which have been reported to decline with aging (Ghodke et al. 2021; Liu et al. 2023). In Group B, we noted an increase in immune‐related genes such as *CD69* and *CCL5* (Blanco‐Dominguez et al. 2022; Scholler et al. 2022), while Group C exhibited heightened expression of cytotoxic genes, including *NKG7*, *CST7*, *IL32*, and *GZMH*, which are linked to the cytotoxicity of CD8^+^ T cells (Wang et al. 2022) (Figure 2c). These findings suggest that the immune response activation observed in Group B accumulates over time, resulting in a significant increase in cytotoxicity in Group C.

Furthermore, we compared DEGs of Group B, Group C, and Group A separately and revealed a 38.1% overlap of DEGs in Group B/A and Group C/A (Figure 2f), highlighting interconnected age‐related molecular changes. The GO term analysis of the shared and upregulated genes were largely associated with T cell receptor binding, positive regulation of T cell activation, antigen processing and presentation of endogenous peptide antigens via MHC class I (Figure 2e). An age‐related alteration observed in CD8^+^ T cells includes increased antigen presentation in human PBMC (Lu et al. 2022). Specifically, CD8^+^ T cells in Group B upregulated human leukocyte antigen *HLA‐A* and *HLA‐E*, with even higher expression levels in Group C, indicating that these alterations begin to manifest significantly in Group B (Figure 2h). Similarly, interferon‐related genes *ISG20* and *ISG15* exhibited increased expression in both Group B and Group C (Figure S2e). In addition, Group B and Group C expressed decreased levels of telomere maintenance (e.g., *CCT5* and *CCT6A*) (Figure S2f). To further explore the degree of age‐related changes between the groups, we calculated the number of DEGs and found that Group C/B had significantly fewer DEGs than the other two groups (Figure S2d). This indicates that the variations between Group B and Group C are less pronounced than those observed in comparisons between the other age groups. Moreover, GO terms of the upregulated genes in Group C/B showed that these genes were largely associated with T cell‐mediated cytotoxicity, including *CD27*, *RHOA*, and *NKG7* (Figure S2g,h). Taken together, our findings indicate that the number of CD8^+^ T cells increases in Group B, accompanied by age‐related changes such as upregulation of T cell activation and antigen‐presenting signaling pathways. Notably, similar changes were also observed in Group C, suggesting that CD8^+^ T cells may contribute to low‐grade inflammation during aging, with these alterations beginning to occur before the typical onset of older age.

### 2.4 Stable Numbers but Declining Function of MRC1^hi^ TRM With Aging

**FIGURE 3:**
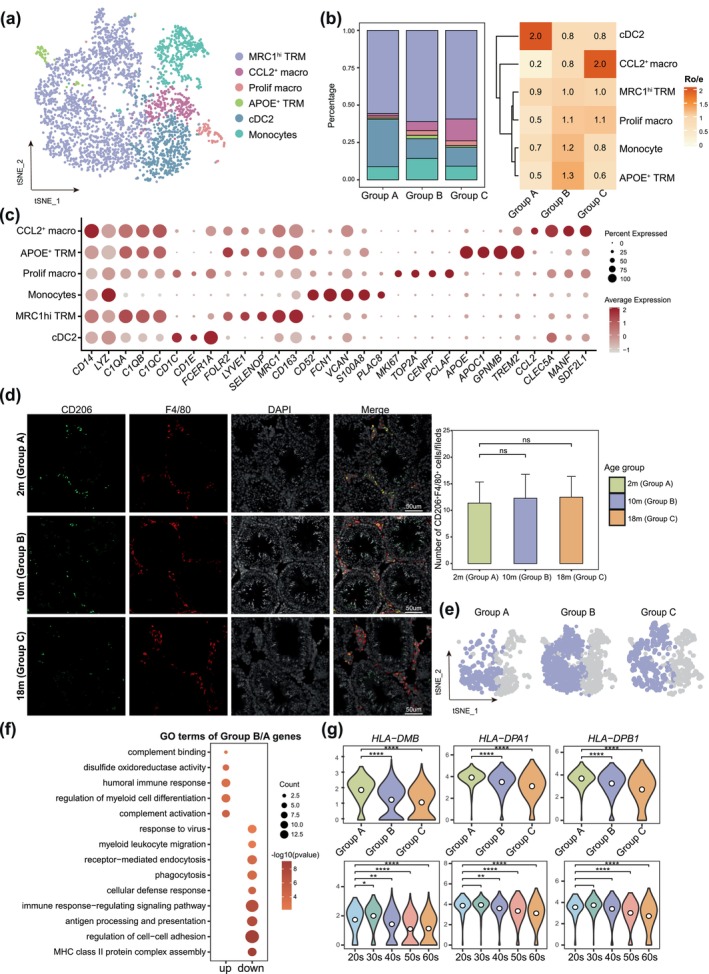
MRC1^hi^ TRM remains relatively stable, with a decline of function during aging. (a) t‐SNE plot showing the myeloid cell subtypes. Color indicates different cell types. (b) Left: Percentage of cell types from (a). Right: Age groups preference for each cell type; Ro/e denotes the ratio of observed to expected cell number. (c) Dot plots showing the expression of marker genes of each cell type from (a). (d) Left: Immunofluorescence (IF) images of MRC1^hi^ TRM marker, CD206 (green), F4/80 (red), across three age groups (2 m [Group A], 10 m [Group B], 18 m [Group C]) in mouse testes. Nuclei were counterstained with DAPI (gray). Right: Quantification of the number of CD206 and F4/80 double‐positive cells in cross sections of fields in groups. Bars represent the mean with SD of 30 independent fields in three age groups (each group *n* = 3). The *p* value was calculated by Student's *t*‐test, ns, no significance. Scale bars, 50 μm. (e) Separate t‐SNE plot of MRC1^hi^ TRM in three age groups. (f) Dot plots showing the GO terms of DEGs of Group B compared to Group A in MRC1^hi^ TRM. (g) Violin plots showing some shared and down‐regulated gene expression in different groups and age groups in MRC1^hi^ TRM. The white dot inside the violin represents the mean. Wilcox test, ****p ≤ 0.0001; ***p ≤ 0.001; **p ≤ 0.01; *p ≤ 0.05.

Myeloid cells are the predominant immune cell types found in both human and mouse testes (Alfano et al. 2021; Jing et al. 2021). To investigate age‐related changes in myeloid cells, we analyzed the myeloid cell clusters depicted in Figure 1c. Upon re‐clustering the myeloid cells based on their unique signature genes, we observed elevated expression levels of marker genes such as *FCN1* and *CD1C*. Our analysis identified various cell types (Figure 3a), including monocytes exhibiting high expression of *FCN1*, *PLAC8*, and *CD52*, as well as testicular resident macrophages (TRM) characterized by the expression of resident genes like *FOLR2*, *LYVE1*, and *SELENOP*. Within the TRM, we distinguished two subtypes: MRC1^hi^ TRM (highly expressing *MRC1* and *CD163*) and APOE^+^ TRM (expressing *APOE*, *GPNMB*, and *TREM2*). Additionally, we identified proliferating macrophages (Prolif macrophages, expressing *TOP2A*, *MKI67*, and *CENPF*), CCL2^+^ macrophages (CCL2^+^ macrophages, expressing *CCL2*, *MANF*, and *SDF2L1*), and type 2 conventional dendritic cells (cDC2 cells, expressing *CD1C*, *CD1E*, and *FCER1A*) (Figure 3c). We further examined the distribution of these cell types across each age group (Figure S3a), confirming the accuracy of our annotations as the marker genes were enriched in pathways corresponding to their specific functions (Figure S3b). Comparative analysis of cell type numbers across the three age groups revealed a significant decrease in the number of cDC2 cells in both Group B and Group C (Figure 3b). In contrast, we observed a marked increase in the two macrophage subtypes and monocytes in Group B, while the most abundant MRC1^hi^ TRM cells remained relatively stable throughout aging (Figure 3b). This finding indicates considerable heterogeneity in the age‐related changes observed in different immune cell types.

Next, we focused on the age‐related changes in MRC1^hi^ TRM. To validate the decline of MRC1^hi^ TRM during aging, we performed immunofluorescence (IF) staining for CD206 (the protein encoded by *MRC1*) and F4/80 (a macrophage marker) in mouse testes at the corresponding time points (Figure 3d). Given the scarcity of healthy human testis tissue samples, we used mice at 2, 10, and 18 months old to represent human ages in Group A, B, and C, respectively. Quantitative analysis showed no significant changes in MRC1^hi^ TRM cell numbers between Group B and C compared to Group A (Figure 3d–e), corroborating our findings.

To explore the molecular changes of MRC1^hi^ TRM during aging, we conducted differential expression analysis between Group B and Group A. Downregulated genes in Group B were enriched in pathways related to phagocytosis (e.g., *FCGR2B*, *CLEC7A*, *CD14*, *APOA1*, *SYK*) and response to virus (e.g., *TLR7*, *BNIP3L*, *NLRP1*) (Figure 3f), consistent with prior studies (McQuattie‐Pimentel et al. 2021; Oishi and Manabe 2016). We calculated signature score of phagocytosis, which revealed significantly lower scores in Groups B and C compared to Group A, indicating a decline in phagocytic function with aging (Figure S3d). Regarding antigen processing and presentation, which was consistent with previously reported (Nie et al. 2022), MRC1^hi^ TRM in Groups B and C upregulated *HLA‐DMB*, *HLA‐DPB1*, and *HLA‐DPA1* (Figure 3g). Notably, the expression of these genes began to decline significantly starting in the 40s, suggesting that age‐related immune changes may initiate in Group B (Figure 3g). Furthermore, upregulated genes in Group B relative to Group A were associated with complement activation (e.g., *C1QA*, *C1QB*, *VSIG4*) and humoral immune response (Figure 3f and Figure S3c). DEG analysis between Groups C and A indicated that both upregulated and downregulated genes were enriched in similar pathways, including phagocytosis and antigen processing and presentation. This suggests that the age‐related changes in MRC1^hi^ TRM begin in Group B and persist with similar trends into Group C (Figure S3e). In summary, our findings demonstrate that the number of MRC1^hi^ TRM in both human and mouse testes remains relatively stable during aging. However, in Groups B and C, we observed a decline in phagocytic and antigen‐presenting functions, along with increased complement activation. This indicates that age‐related immune alterations may commence during Group B, highlighting the need for further investigation into the implications of these changes for testicular immune function and overall health in aging individuals.

### 2.5 Monocytes Accumulation in Testes and Potential Contribution to Low‐Degrade Inflammation

**FIGURE 4:**
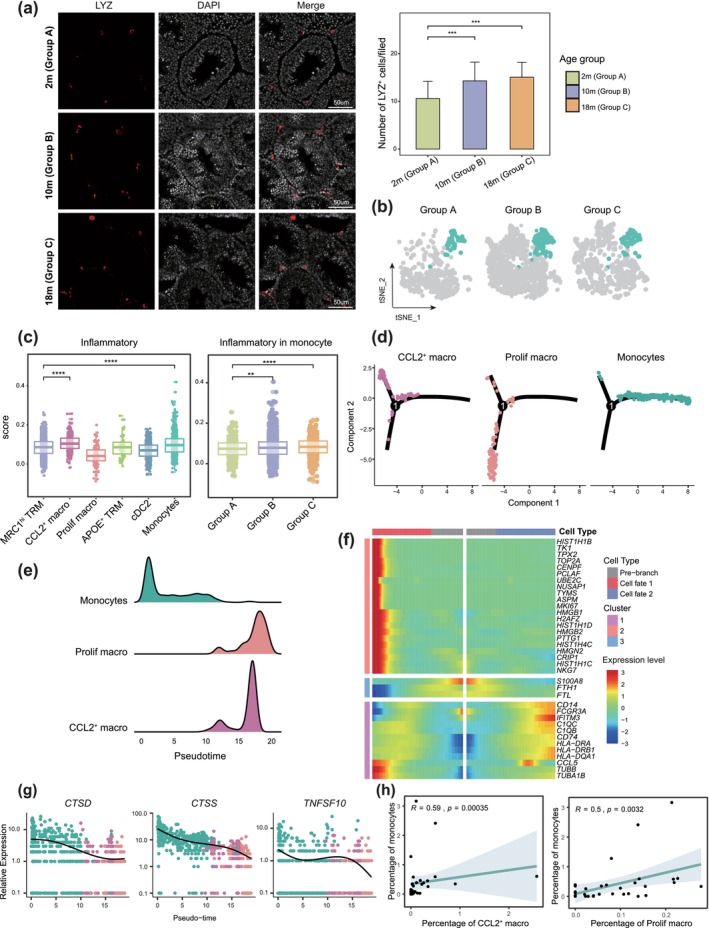
Monocytes accumulated and contributed to low‐grade inflammation during aging. (a) Left: IF staining of LYZ (red) in tissue sections of three groups (2 m [Group A], 10 m [Group B], 18 m [Group C]) in mice testes. Nuclei were counterstained with DAPI (grey). Right: Quantification of the number of positive LYZ cells in cross sections of fields in different groups. Bars represent the mean with SD of 30 independent fields in three age groups (each group *n* = 3). The *p* value was calculated by Student's *t*‐test, ***p ≤ 0.001. Scale bars, 50 μm. (b) Separate t‐SNE plot of monocytes in three age groups. (c) Left: Box plot showing the signature score of the inflammatory response of myeloid subtypes. Right: The signature score of the inflammatory response of monocytes in three age groups. Box indicates the range from the 25th to the 75th percentile; center lines indicate median, dot represents individual cells. The *p* value was calculated by Student's *t*‐test. ****p ≤ 0.0001; **p ≤ 0.01. (d) Trajectories of CCL2^+^ macrophages, Prolif macrophages, and monocytes by pseudotime analysis. (e) The cell distribution of CCL2^+^ macrophages, Prolif macrophages, and monocytes, along with the pseudotime, is color‐coded by cell types. (f) Heatmap illustrating the differentially expressed genes toward cell state 1 and cell state 2 along the pseudotime trajectories. (g) The dynamic expression of *CTSD*, *CTSS*, and *TNFSF10* along the pseudotime trajectory. (h) The correlations between the percentage of monocytes and CCL2^+^ macrophages (left) and Prolif macrophages (right).

Monocytes play a critical role in inflammation during aging (Cao et al. 2022; Hearps et al. 2012; Kim and Benayoun 2021). We identified monocytes and observed a significant increase in number in Group B (Figure 3b). This finding was further supported by IF staining, which showed a marked increase in LYZ‐positive cells (a monocytes marker) in the testes of mice in Groups B and C (Figure 4a). Inflammatory response scoring for myeloid subtypes revealed elevated scores in monocytes and CCL2^+^ macrophages in Groups B and C compared to Group A (Figure 4c), indicating that monocytes exhibit pro‐inflammatory features and likely contribute to low‐grade inflammation with aging.

To further explore the relationship between monocytes and macrophage subtypes, we performed pseudotime analysis for monocytes, CCL2^+^ macrophages, and Prolif macrophages to infer developmental trajectories (Figure S4a). Pseudotime analysis placed monocytes at the origin, diverging into two distinct branches—CCL2^+^ and Prolif macrophages—indicating their differentiation into functionally specialized subtypes (Figure 4d). This pattern suggests that monocytes may evolve into these macrophage subtypes later in the trajectory (Figure 4e). Distinct expression patterns were shown with immune regulatory genes such as *CCL5*, *IFITM3*, *FCGR3A*, and *CD74*, which were highly expressed in cell state 2 and downregulated in cell state 1 (Figure 4f). Notably, *IFITM3* is associated with the immune response to pathogens (Hur et al. 2020) while *CD74* is involved in the recruitment and migration of immune cells (Qin et al. 2023). We further investigated the transcriptional alterations correlated with transitional states; the expression levels of *CTSD*, *CTSS*, and *TNFSF10* were higher in monocytes (Figure 4g). *CTSS* and *CTSD*, members of the cathepsin family, are crucial mediators of inflammation (Wan et al. 2023). In addition, inflammatory genes such as *SPP1*, *MAF*, and *MSR1* showed elevated expression in CCL2^+^ and Prolif macrophages, indicating their potential role in sustaining the low‐grade inflammation linked to aging (Figure S4c).

To clarify the relationships among CCL2^+^ macrophages, Prolif macrophages, and monocytes during aging, we performed cell proportion and Pearson correlation analyses, revealing a strong positive correlation between monocytes and both macrophage subtypes (Figure 4h). Together, these findings suggest that the increased infiltration of monocytes in aging testes may drive the differentiation of CCL2^+^ and Prolif macrophages, contributing to the low‐grade inflammation characteristic of aging.

### 2.6 Age‐Related Inflammatory Activation in Monocytes

**FIGURE 5:**
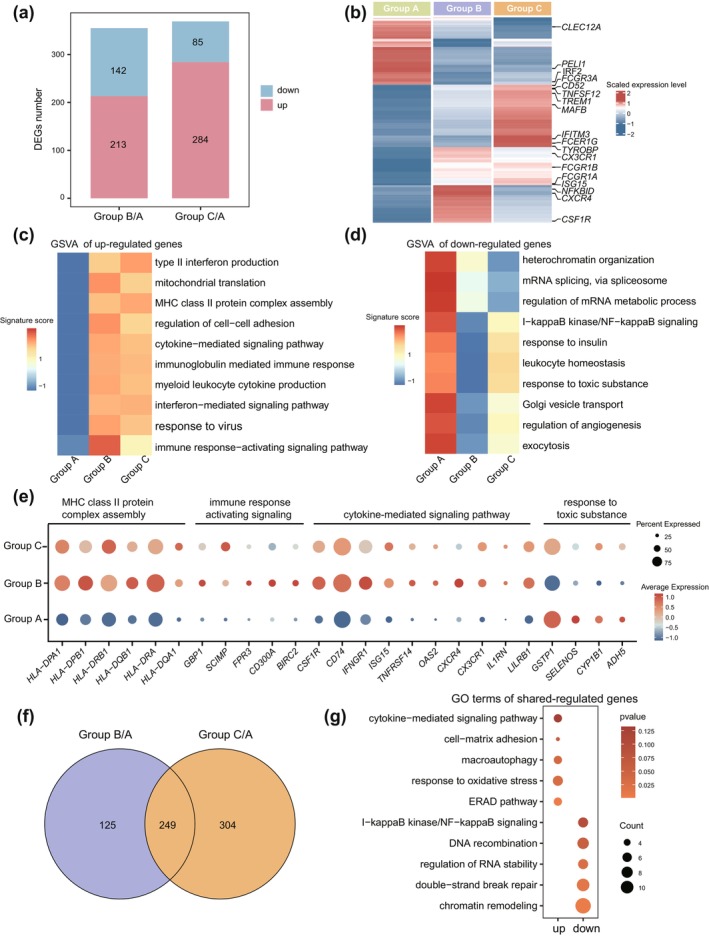
Activated immune signaling of monocytes in Group B persists during aging. (a) Bar plots showing the DEG number of monocytes in Group B/A, Group C/A. Red (or blue) represents the upregulated (or downregulated) DEGs. (b) Heatmap showing the DEGs of each age group in monocytes. The scaled gene expression levels are colored according to *Z*‐score. (c) Heatmap showing the enrichment of upregulated genes of monocytes in Groups B and C by GSVA analysis. The enriched pathways were on the right side. Color indicates signature score. (d) Heatmap showing the enrichment of downregulated genes of monocytes in Groups B and C by GSVA analysis. The enriched pathways were on the right side. Color indicates signature score. (e) Dot plot showing the expression of genes in the pathways from (c) and (d) in three age groups. The enriched pathways were listed at the top. Color represents the average expression. (f) Venn diagrams showing the number of significantly differentially expressed genes in CCL2^+^ macrophages and their overlap. (g) Representative GO terms enrichment of overlap genes from (f) and their associated *p* value.

Our analysis revealed significant age‐related changes in monocytes and their interactions with other immune cells. Differential expression analysis indicated similar numbers of DEGs in Group B/A and Group C/A (Figure 5a). Specifically, genes such as *TREM1*, *MAFB*, *IFITM3*, and *ISG15* were upregulated in Group B and showed further increased expression in Group C (Figure 5b). To further investigate functional changes in monocytes during aging, we conducted Gene Set Variation Analysis (GSVA) across age groups, revealing that immune pathways, including type II interferon production, cytokine signaling, and immune response activation, displayed higher scores in both Group B and Group C (Figure 5c). Notably, genes involved in MHC class II protein complex assembly (e.g., *HLA‐DPA1*, *HLA‐DPB1*, *HLA‐DRB1*, *HLA‐DRA*) were upregulated in Group B and Group C (Figure 5e), which is contrary to age‐related changes observed in MRC1^hi^ TRM (Figure 3g). Cytokine‐related genes like *ISG15*, *TNFRSF14*, and *IL1RN* also showed increased expression, indicating a shift toward a pro‐inflammatory state (Munnur et al. 2021). Downregulated genes in Group B and C were enriched in pathways related to mRNA metabolic regulation, Golgi vesicle transport, and exocytosis (Figure 5d), suggesting common age‐related changes in monocytes. In summary, monocytes demonstrated increased expression of inflammatory genes and enhanced inflammatory functions with aging, potentially driving low‐grade inflammation.

### 2.7 Age‐Related Inflammatory Activation in CCL2 ^+^ Macrophages and cDC2 Cells

CCL2^+^ macrophages displayed increased immune infiltration during aging (Figure 3b). Investigating the molecular changes in these cells revealed substantial overlap in DEGs between Group B/A and Group C/A, indicating shared age‐related molecular signatures (Figure 5f). GO analysis of shared and upregulated genes pointed to enrichment in cytokine‐mediated signaling, oxidative stress response, and the ERAD (endoplasmic reticulum‐associated degradation) pathway, while downregulated genes were associated with DNA recombination and chromatin remodeling (Figure 5g). To determine whether these molecular changes were consistent across age groups, we examined 304 DEGs unique to Group C. Upregulated genes in Group C were enriched in interleukin‐6 production and type II interferon response, while downregulated genes involved pattern recognition receptor signaling and chromatin remodeling (Figure S5a). The expression of the classic pattern recognition receptor, *TLR4*, and *MYD88* (Munnur et al. 2021), declined significantly in Group C, with earlier reductions starting in Group B (Figure S5b).

In addition, cDC2 cells, essential for coordinating immune protection and response to extracellular pathogens through antigen presentation (Rodrigues et al. 2023), showed a marked age‐related decline, beginning in Group B (Figure 3b) and consistent with previous PBMC aging studies (Cui et al. 2024). GO analysis of DEGs between Group B and Group A highlighted downregulation in pathways for viral response and mRNA stability (Figure S5c). Age‐specific expression patterns were observed in MHC class II molecules, with *HLA‐DRB1* and *HLA‐DQA1* upregulated, while *HLA‐DQA2* and *HLA‐DMB* were downregulated (Figure S5d). Furthermore, genes related to immune response signaling and myeloid cell differentiation were enriched in both Group B/A and Group C/A (Figure S5e‐f). Overall, these findings suggest that age‐related changes in cDC2 cells likely begin in Group B and accumulate in Group C.

## 3 Discussion

Low‐grade inflammation is a hallmark of aging, making it essential to understand how immune cells change over time to uncover the mechanisms driving tissue aging. In males, testis aging contributes to declines in fertility and testosterone levels, compromising both reproductive and overall health (Kaufman et al. 2019; Santiago et al. 2019). While previous research has primarily focused on the changes in somatic cells and germ cells within the aging testis (Xia et al. 2024), recent studies also show that age‐related alterations in testicular somatic cells include the upregulation of inflammation‐related genes. Immune cells are central regulators of the testicular immune environment, playing critical roles in testis development, tissue repair, and the pathophysiological processes underlying testis aging (Zhao et al. 2014). For instance, regulatory T cells (Tregs) maintain the tolerogenic environment of the testis, and its depletion in mice leads to reduced sperm number and motility, causing severe fertility defects (Barrachina et al. 2023). Moreover, inhibiting macrophage activation offers a strategy to mitigate chronic inflammation associated with testicular in aging mice (Chi et al. 2024). Despite these insights, the heterogeneity of immune cells and their age‐related changes remain largely unknown, particularly in humans. In this study, we applied scRNA‐seq analysis to 33 human testis samples, effectively mapping the age‐related alterations in immune cell populations.

Our findings reveal distinct shifts in immune cell subtypes as individuals age: CD8^+^ T cells increase, while myeloid cells decrease, cDC2 cells show a significant decline, MRC1^hi^ TRM cells remain stable, and monocytes increase. These results indicate that the immune microenvironment within the testis undergoes dynamic and significant changes with aging, shedding light on its potential role in testicular aging. Furthermore, this study looked into the potential molecular inferences underlying these alterations, highlighting their implications for understanding testicular aging and how immune system changes contribute to age‐related testicular dysfunction.

By categorizing individuals aged 21–69 years, we observed that Group B (aged 40–50) and Group C (aged 50–69) exhibit similar ‐related changes compared to Group A (aged 21–38), which align with alterations noted in PBMC (Shen et al. 2024; Simnica et al. 2019). Notably, CD8^+^ T cells in Groups B and C demonstrated higher expression levels of MHC class I and chemokines compared to Group A, paralleling the molecular and functional changes documented in CD8^+^ T cells in PBMC (Lu et al. 2022; Terrabuio et al. 2023). Moreover, T cells in the ovaries of middle‐aged women similarly exhibit increased features of senescence, aligning with the period of age‐related changes observed in the testes (Wu et al. 2024). Our analysis revealed that MRC1^hi^ TRM cells maintained relatively stable numbers during aging in both human and mouse testes; however, these cells exhibited age‐related functional changes, such as alterations in antigen presentation and phagocytosis, which are consistent with documented age‐related changes in macrophages (Kelly et al. 2007; Kotter et al. 2006; Natrajan et al. 2015; Wang, Hong, et al. 2024). The increased infiltration of monocytes has been linked to various inflammatory conditions, including aging and orchitis (Barman et al. 2022; Orozco et al. 2021; Shi and Pamer 2011; Wang et al. 2021). A previous study showed that inflammatory testicular macrophages, mainly from circulating monocytes, promoted testis damage and adversely affect spermatogenesis (Wang et al. 2021). Our study found an increase in monocytes in both human and mouse testes. Pseudotime analysis further revealed their potential to differentiate into CCL2^+^ macrophages and Prolif macrophages, with monocytes percentages positively correlating with those of these macrophage subtypes. Additionally, during aging, monocytes exhibited heightened activation of immune responses, characterized by increased cytokine‐mediated signaling and type II interferon production. This suggests a role for monocytes in promoting the low‐grade inflammation associated with testicular aging. Our findings indicate that molecular and cellular changes related to aging begin in Group B and persist into Group C, aligning with the aging patterns observed in PBMC (Cui et al. 2024; Rodier et al. 2009; Wong and Goldstein 2013). This suggests that the aging patterns of testicular immune cells mirror those of PBMC, implying that systemic immunity aging may influence the age‐associated changes within the testicular immune microenvironment.

Immune cells are pivotal in various male spermatogenic disorders (Bhushan et al. 2020; Yang et al. 2023; Yang et al. 2024; Zheng et al. 2021). Therefore, comprehending the types of immune cells present in the testis is crucial for elucidating the mechanisms underlying testicular development and associated diseases. For the first time, we identified monocytes and cDC2 cells in the human testis and observed cellular and molecular changes related to aging. In mouse testes, macrophages are classified as peritubular and interstitial macrophages (Gu et al. 2023; Lokka et al. 2020). The alterations observed in MRC1^hi^ TRM from our human testicular single‐cell data partially reflect the cellular and molecular changes seen in interstitial macrophages during aging. Furthermore, the age‐related changes observed in MRC1^hi^ TRM and monocytes in human testes align with findings in mouse testis, indicating that these alterations are conserved across species. This conservation facilitates further exploration of testicular aging biomarkers and interventions using model organisms. Nevertheless, the mechanisms underlying these age‐related changes remain inadequately understood, particularly concerning the identification of biomarkers for aging in testicular immune cells and how shifts in phagocytic activity and cytokine production in these cells impact germ cells and somatic cells. Further investigation is warranted to clarify their roles in testicular aging and related disorders.

In conclusion, our comprehensive analysis of large‐cohort human testicular immune cells provides valuable data for scientific research. This study elucidates the molecular changes and presents a detailed atlas of the aging profile of human testicular immune cells. Moreover, the similarities in aging trends between human and mouse testicular immune cells enhance the utility of animal models in investigating the mechanisms of testicular aging. These findings offer critical insights into male reproductive health and underscore the importance of understanding immune cell dynamics within the context of aging.

## 4 Methods

### 4.1 Sample Inclusion

Samples were included from a publicly available scRNA‐seq study (GEO: GSE254315) of testicular tissue from adult males and include 12 samples from a previous study (GEO: GSE182786). We used 33 samples of these, ranging in age from 21 to 69 years. Specifically, all tissue processing and sequencing were conducted by the same technician using the same sequencing platform, ensuring technical consistency.

### 4.2 Processing of Single Cell RNA‐Seq Data

Download the processed data from GEO. Use the Read10X function in Seurat (https://satijalab.org/seurat/index.html, R package, v4.3.1) to sequentially load matrices into R and add sample information to the row names of the matrix; all this information was merged to create a Seurat object using the merge function. The data has undergone filtering and normalization. Features with fewer than 300 or more than 8000 features, UMIs with fewer than 300 or more than 70,000 counts, and cells with > 20% mitochondrial mapping were filtered out. Subsequently, the integration of the datasets exhibited little batch effect in different ages. PCA and t‐SNE analysis were performed based on 3000 high variable genes (HVGs) and 1–25 PCs. Identification of cell types was performed based on the tutorial. When analyzing individual cell subpopulations, the subset function was used to extract cells based on cell type annotations and form new Seurat objects. The Seurat objects of Prolif macrophages, CCL2^+^ macrophages, and monocytes were converted into CellDataSet objects for importing into the Monocle package (v2.28.0). Branch expression analysis modeling (BEAM) was performed to find genes that were regulated in a branch‐dependent way.

### 4.3 Differentially Expressed Genes and Functional Enrichment Analysis

We divided ages into 20s, 30s, 40s, 50s, and 60s, and also divided into Group A (< 40 years old), Group B (40–50 years old), and Group C (> 50 years old). Differential gene expression analysis was performed using the FindMarkers function in Seurat Stuart et al. 2019. Genes were considered significant if the *p* value was < 0.05 and the Log_2_ fold change (log_2_FC) was > 0.5 or < −0.5. Significant DEGs for each cluster and groups were used as input for Gene Ontology (GO) analysis by ClusterProfiler Wu et al. 2021. We selected the *p* value < 0.05 and gene count > 3 as significant pathways. When scoring special enriched functions or pathways enriched in GO analysis, the AddModuleScore function from the Seurat package was used to calculate the average expression levels of each age group on single‐cell level based on the genes enriched in the special enriched functions or pathways.

To obtain the differentially expressed gene sets between age groups, GSVA Hänzelmann et al. 2013 (R package GSVA) was performed. GSVA ranked all the genes in the dataset based on DEGs from age groups.

To visualize the result of difference analysis, ggvenn (R package, v0.1.10), ggpubr (R package, v0.6.0), ComplexHeatmap (R package, v2.16.0), and ggplot2 (R package, v3.5.1) were used.

### 4.4 Immunofluorescence Staining

The male wild‐type C57BL/6 mice were purchased from Yantai Jiahe Medical Technology Co. LTD. Testes of 2, 10, and 18‐month‐old mice were dissected in PBS and fixed overnight at 4°C in animal testicular tissue fixative (servicebio, G1121); after overnight fixation, testes were processed through a sucrose: PBS gradient (10%, 15%, 20% sucrose) and were embedded in OCT medium (Tissue‐Tek, cat. 4583) at −80°C prior to cryosectioning. After cryosectioning, samples were soaked overnight at 4°C in PBST (PBS + 0.1% Tween‐20). Remove and rewarm for half an hour, wash with PBS on a shaking table for 3 × 5 min. After that, it was used for about 250 mL of 1×Tris‐EDTA (PH 9.0, supplemented with Tween 20 to 0.05%) antigen repair solution, heated in a plastic dye box in a water bath at 95°C for about 15 min, and then put the slices into the box and repaired for 15 min, and remove the box and allow to cool naturally at room temperature, wash with PBS on a shaking table for 3 × 5 min. Add 50 μL of 5% BSA/PBS to the surface of the tissue, and close the wet box at room temperature for 30 min. Dilute the primary antibody to the corresponding concentration with the closure solution and then add 50 μL of the primary antibody, including anti‐LYZ (ab108508, abcam, 1:300), anti‐F4/80 (28963‐1‐AP, proteintech, 1:2000), and anti‐CD206 (GB113497, servicebio, 1:400) to the surface of the tissue, and hybridize in the wet box at 4°C overnight. Remove and rewarm for half an hour, wash with PBST on a shaking table for 3 × 5 min, then dilute the secondary antibody with PBST to the corresponding concentration and add 50 μL to the surface of the tissue and hybridize in the wet box at room temperature for 2 h. Wash with PBST on a shaking table for 3 × 5 min. After washing, the liquid on the slide surface was blotted out, and 20 μL of sealer (containing DAPI) was added to the surface of the tissue, a coverslip was applied, and the slides were sealed with nail polish. The co‐staining of anti‐F4/80 and anti‐CD206 was performed following the four‐color multifluorescent immunohistochemical staining kit (Pika Universal Secondary Antibody) (pH 9.0) (abs50012, abisin).

### 4.5 Cell Counting and Statistical Analysis

All the images were obtained by Zeiss LSM880. To quantify CD206^+^F4/80^+^ cells, double‐positive cells were counted, while LYZ^+^ cells were quantified by counting single‐positive cells. Each sample selected 10 fields for positive staining cell count, each group of 3 samples. Quantitative data were analyzed using Student's *t‐*test. n.s., not significant; *p < 0.05; **p < 0.01; ***p < 0.001; ****p < 0.0001 were considered statistically significant.

## Author Contributions

Jingtao Guo conceived the study. Xiaoyan Wang oversaw the overall study. Qiaoling Jiang performed computational data analysis. Lina Cui, Xiaojian Lu, and Bradley R. Cairns assisted with data interpretation. Xichen Nie, Hui Cai, Yifei Guo, Wenxiu Zhang, and James M. Hotaling performed histological experiments and confocal photography.

## Conflicts of Interest

The authors declare no conflicts of interest.

## Supporting information


**Figure S1**Single‐cell analysis of immune cells in human testis during aging. (a) Heatmap showing the correlation between samples. The color scale represents the correlation. (b) Separate t‐SNE plot of major testicular cell types in groups. SMC, smooth muscle cells. (c) Expression of selected markers identifying major testicular cell types. Expression level as shown on the color at the right. (d) Bar plot showing the percentage of Sertoli cells in each group. (e) Violin plots showing the UMIs/genes/percent.mito in overall cells from left to right.

**Figure S2**Age‐related features increased in Group B. (a) Bar plot showing the value of coefficient of variation (cv) of immune cells. Each group randomly selected 3 samples (*n* = 3). Box indicates range from 25th to 75th percentile; center lines indicate medium. The *p* value was calculated by Student’s *t*‐test. ****p < 0.0001. (b) Bar plot showing the percentage of immune subtypes in three age groups. (c) Line plot showing the percentage of CD8^+^ T cells in immune cells among three age groups. Color indicates each age group. (d) The DEGs number of CD8^+^ T cells in Group B/A, C/A and C/B. (e) Violin plots showing the expression of genes in negative regulation of viral genome replication from (Figure 2g). (f) Violin plots showing the expression of genes in telomere maintenance from (Figure 2g). (g) Representative GO terms enrichment of DEGs in Group C/B from (d) and their associated *p* value. (h) Dot plots showing the selected genes of pathways from (g).

**Figure S3**MRC1^hi^ TRM remain relatively stable, with a decline of function during aging. (a) Separate t‐SNE plot of myeloid subtypes in each age group. (b) Left: heatmap showing the top 100 differentially expressed genes of each cell cluster from (Figure 3a). The scaled gene expression levels are colored according to *Z* score. Right: the corresponding top two GO terms enriched in the marker genes of each cell cluster with *Z* score colored according to the color key at the right. (c) Dot plot showing the expression of genes in the pathways from (Figure 3f) in three age groups. The enriched pathway on the top. Color represents the average expression. (d) Box plot showing the signature score of phagocytosis of monocytes among three age groups. Color indicates the age groups, box indicates range from 25th to 75th percentile; center lines indicate medium, dot represent individual cells. The *p* value was calculated by Student’s *t*‐test. **p ≤ 0.01, *p ≤ 0.05. (e) Dot plots showing the GO terms of DEGs of Group A to Group B in MRC1^hi^ TRM.

**Figure S4**Monocytes accumulated and contributed low‐grade inflammation during aging. (a) Heatmap showing the correlations of each myeloid subtypes. (b) CCL2^+^ macrophages, Prolif macrophages and monocytes pseudotime analysis using Monocle2. (c) The dynamic expression of *GPR34*, *MAF*, *MSR1*, *NEAT1*, *SPP1* along the pseudotime trajectory.

**Figure S5** Age‐related changes in cDC2 cells. (a) Representative GO terms enrichment of 304 unique DGEs of Group C/A from (Figure 5f) and their associated *p* value. (b) Violin plots showing the expression level of selected genes from (a) in CCL2^+^ macrophages. The white dot inside the violin represents the mean. Wilcox.test, ****p ≤ 0.0001; *p ≤ 0.05; ns, *p* > 0.05. (c) Representative GO terms enrichment of DEGs in Group B/A in cDC2 cells and their associated *p* value. (d) Violin plots showing the expression level of selected genes from (c) The white dot inside the violin represents the mean. Wilcox.test,  ; ***p ≤ 0.001; **p ≤ 0.01; *p ≤ 0.05; ns, *p* > 0.05. (e) Representative GO terms enrichment of DEGs in Group C/A in cDC2 cells and their associated *p* value. (f) Violin plots showing the expression level of selected genes from (e). The white dot inside the violin represents the mean. Wilcox.test, ; ***p ≤ 0.001; **p ≤ 0.01; *p ≤ 0.05; ns, *p* > 0.05.

## Data Availability

Single‐cell sequencing data can be found in NCBI GEO under accession number GSE254315, which includes 12 datasets from GSE182786.

## References

[acel70032-bib-0001] Alfano, M. , A. S. Tascini , F. Pederzoli , et al. 2021. “Aging, Inflammation and DNA Damage in the Somatic Testicular Niche With Idiopathic Germ Cell Aplasia.” Nature Communications 12, no. 1: 5205. 10.1038/s41467-021-25544-0. PMC8410861 34471128

[acel70032-bib-0002] Barman, P. K. , J. E. Shin , S. A. Lewis , et al. 2022. “Production of MHCII‐Expressing Classical Monocytes Increases During Aging in Mice and Humans.” Aging Cell 21, no. 10: e13701. 10.1111/acel.13701. 36040389 PMC9577948

[acel70032-bib-0003] Barrachina, F. , K. Ottino , M. L. Elizagaray , et al. 2023. “Regulatory T Cells Play a Crucial Role in Maintaining Sperm Tolerance and Male Fertility.” Proceedings of the National Academy of Sciences of the United States of America 120, no. 37: e2306797120. 10.1073/pnas.2306797120. 37676910 PMC10500189

[acel70032-bib-0004] Bhushan, S. , M. S. Theas , V. A. Guazzone , et al. 2020. “Immune Cell Subtypes and Their Function in the Testis.” Frontiers in Immunology 11: 583304. 10.3389/fimmu.2020.583304. 33101311 PMC7554629

[acel70032-bib-0005] Blackburn, E. H. , E. S. Epel , and J. Lin . 2015. “Human Telomere Biology: A Contributory and Interactive Factor in Aging, Disease Risks, and Protection.” Science 350, no. 6265: 1193–1198. 10.1126/science.aab3389. 26785477

[acel70032-bib-0006] Blanco‐Dominguez, R. , H. de la Fuente , C. Rodriguez , et al. 2022. “CD69 Expression on Regulatory T Cells Protects From Immune Damage After Myocardial Infarction.” Journal of Clinical Investigation 132, no. 21: 2418. 10.1172/JCI152418. PMC9621142 36066993

[acel70032-bib-0007] Cao, Y. , Y. Fan , F. Li , et al. 2022. “Phenotypic and Functional Alterations of Monocyte Subsets With Aging.” Immunity & Ageing 19, no. 1: 63. 10.1186/s12979-022-00321-9. 36514074 PMC9745938

[acel70032-bib-0008] Carrasco, E. , M. M. Gomez de Las Heras , E. Gabande‐Rodriguez , G. Desdin‐Mico , J. F. Aranda , and M. Mittelbrunn . 2022. “The Role of T Cells in Age‐Related Diseases.” Nature Reviews Immunology 22, no. 2: 97–111. 10.1038/s41577-021-00557-4. 34099898

[acel70032-bib-0009] Chambers, E. S. , M. Vukmanovic‐Stejic , B. B. Shih , et al. 2021. “Recruitment of Inflammatory Monocytes by Senescent Fibroblasts Inhibits Antigen‐Specific Tissue Immunity During Human Aging.” Nature Aging 1, no. 1: 101–113. 10.1038/s43587-020-00010-6. 37118005

[acel70032-bib-0010] Chi, A. , B. Yang , H. Dai , et al. 2024. “Stem Leydig Cells Support Macrophage Immunological Homeostasis Through Mitochondrial Transfer in Mice.” Nature Communications 15, no. 1: 2120. 10.1038/s41467-024-46190-2. PMC10924100 38459012

[acel70032-bib-0011] Cui, Q. , W. Li , D. Wang , et al. 2024. “Immune Signature and Phagocytosis of Circulating DC Subsets in Healthy Adults During Aging.” International Immunopharmacology 130: 111715. 10.1016/j.intimp.2024.111715. 38382263

[acel70032-bib-0090] Cui, L. , X. Nie , Y. Guo , et al. Single‐cell transcriptomic atlas of the human testis across the reproductive lifespan. Nat Aging. 2025 Mar 3. 10.1038/s43587-025-00824-2. Epub ahead of print. PMID: 40033047. PMC12003174 40033047

[acel70032-bib-0012] De Silva, N. S. , J. Siewiera , C. Alkhoury , et al. 2023. “Nuclear Envelope Disruption Triggers Hallmarks of Aging in Lung Alveolar Macrophages.” Nature Aging 3, no. 10: 1251–1268. 10.1038/s43587-023-00488-w. 37723209

[acel70032-bib-0013] Diao, D. , H. Wang , T. Li , et al. 2018. “Telomeric Epigenetic Response Mediated by Gadd45a Regulates Stem Cell Aging and Lifespan.” EMBO Reports 19, no. 10: 5494. 10.15252/embr.201745494. PMC6172461 30126922

[acel70032-bib-0014] Endo, T. , K. Kobayashi , T. Matsumura , et al. 2024. “Multiple Ageing Effects on Testicular/Epididymal Germ Cells Lead to Decreased Male Fertility in Mice.” Communications Biology 7, no. 1: 16. 10.1038/s42003-023-05685-2. 38177279 PMC10766604

[acel70032-bib-0015] Franceschi, C. , P. Garagnani , P. Parini , C. Giuliani , and A. Santoro . 2018. “Inflammaging: A New Immune‐Metabolic Viewpoint for Age‐Related Diseases.” Nature Reviews Endocrinology 14, no. 10: 576–590. 10.1038/s41574-018-0059-4. 30046148

[acel70032-bib-0016] Ghodke, I. , M. Remisova , A. Furst , et al. 2021. “AHNAK Controls 53BP1‐Mediated p53 Response by Restraining 53BP1 Oligomerization and Phase Separation.” Molecular Cell 81, no. 12: 2596–2610. 10.1016/j.molcel.2021.04.010. 33961796 PMC8221568

[acel70032-bib-0017] Groh, J. , K. Knopper , P. Arampatzi , et al. 2021. “Accumulation of Cytotoxic T Cells in the Aged CNS Leads to Axon Degeneration and Contributes to Cognitive and Motor Decline.” Nature Aging 1, no. 4: 357–367. 10.1038/s43587-021-00049-z. 37117598

[acel70032-bib-0018] Gu, X. , A. Heinrich , S. Y. Li , and T. DeFalco . 2023. “Testicular Macrophages Are Recruited During a Narrow Fetal Time Window and Promote Organ‐Specific Developmental Functions.” Nature Communications 14, no. 1: 1439. 10.1038/s41467-023-37199-0. PMC10017703 36922518

[acel70032-bib-0019] Han, G. , S. H. Hong , S. J. Lee , S. P. Hong , and C. Cho . 2021. “Transcriptome Analysis of Testicular Aging in Mice.” Cells 10, no. 11: 2895. 10.3390/cells10112895. 34831115 PMC8616291

[acel70032-bib-0082] Hänzelmann, S. , R. Castelo , and J. Guinney . 2013. “GSVA: Gene Set Variation Analysis for Microarray and RNA‐Seq Data.” BMC Bioinformatics 14, no. 1. 10.1186/1471-2105-14-7. PMC3618321 23323831

[acel70032-bib-0020] Hasan, H. , W. Peng , R. Wijayarathna , et al. 2024. “Monocytes Expressing Activin A and CCR2 Exacerbate Chronic Testicular Inflammation by Promoting Immune Cell Infiltration.” Human Reproduction 39: 1404–1422. 10.1093/humrep/deae107. 38775335

[acel70032-bib-0021] He, M. , Y. Han , C. Cai , et al. 2021. “CLEC10A Is a Prognostic Biomarker and Correlated With Clinical Pathologic Features and Immune Infiltrates in Lung Adenocarcinoma.” Journal of Cellular and Molecular Medicine 25, no. 7: 3391–3399. 10.1111/jcmm.16416. 33655701 PMC8034442

[acel70032-bib-0022] Hearps, A. C. , G. E. Martin , T. A. Angelovich , et al. 2012. “Aging Is Associated With Chronic Innate Immune Activation and Dysregulation of Monocyte Phenotype and Function.” Aging Cell 11, no. 5: 867–875. 10.1111/j.1474-9726.2012.00851.x. 22708967

[acel70032-bib-0023] Hou, J. , K. X. Chen , C. He , et al. 2024. “Aged Bone Marrow Macrophages Drive Systemic Aging and Age‐Related Dysfunction via Extracellular Vesicle‐Mediated Induction of Paracrine Senescence.” Nature Aging 4: 1562–1581. 10.1038/s43587-024-00694-0. 39266768 PMC11564114

[acel70032-bib-0024] Huang, D. , Y. Zuo , C. Zhang , et al. 2023. “A Single‐Nucleus Transcriptomic Atlas of Primate Testicular Aging Reveals Exhaustion of the Spermatogonial Stem Cell Reservoir and Loss of Sertoli Cell Homeostasis.” Protein & Cell 14, no. 12: 888–907. 10.1093/procel/pwac057. 36929025 PMC10691849

[acel70032-bib-0025] Hur, J. Y. , G. R. Frost , X. Wu , et al. 2020. “The Innate Immunity Protein IFITM3 Modulates Gamma‐Secretase in Alzheimer's Disease.” Nature 586, no. 7831: 735–740. 10.1038/s41586-020-2681-2. 32879487 PMC7919141

[acel70032-bib-0026] Jing, Y. , M. Cao , B. Zhang , X. Long , and X. Wang . 2021. “cDC1 Dependent Accumulation of Memory T Cells Is Required for Chronic Autoimmune Inflammation in Murine Testis.” Frontiers in Immunology 12: 651860. 10.3389/fimmu.2021.651860. 34381443 PMC8350123

[acel70032-bib-0027] Kaufman, J. M. , B. Lapauw , A. Mahmoud , G. T'Sjoen , and I. T. Huhtaniemi . 2019. “Aging and the Male Reproductive System.” Endocrine Reviews 40, no. 4: 906–972. 10.1210/er.2018-00178. 30888401

[acel70032-bib-0028] Kaya, T. , N. Mattugini , L. Liu , et al. 2022. “CD8(+) T Cells Induce Interferon‐Responsive Oligodendrocytes and Microglia in White Matter Aging.” Nature Neuroscience 25, no. 11: 1446–1457. 10.1038/s41593-022-01183-6. 36280798 PMC9630119

[acel70032-bib-0029] Kelly, J. , A. Ali Khan , J. Yin , T. A. Ferguson , and R. S. Apte . 2007. “Senescence Regulates Macrophage Activation and Angiogenic Fate at Sites of Tissue Injury in Mice.” Journal of Clinical Investigation 117, no. 11: 3421–3426. 10.1172/JCI32430. 17975672 PMC2045608

[acel70032-bib-0030] Kim, M. , and B. A. Benayoun . 2021. “A Multiomic Atlas for the Exploration of Healthy Aging in Human Monocytes.” Nature Aging 1, no. 1: 19–21. 10.1038/s43587-020-00007-1. 37117994

[acel70032-bib-0031] Korbecki, J. , M. Bosiacki , P. Kupnicka , K. Barczak , D. Chlubek , and I. Baranowska‐Bosiacka . 2024. “CXCR4 as a Therapeutic Target in Acute Myeloid Leukemia.” Leukemia 38, no. 11: 2303–2317. 10.1038/s41375-024-02326-3. 39261603

[acel70032-bib-0032] Kotter, M. R. , W. W. Li , C. Zhao , and R. J. Franklin . 2006. “Myelin Impairs CNS Remyelination by Inhibiting Oligodendrocyte Precursor Cell Differentiation.” Journal of Neuroscience 26, no. 1: 328–332. 10.1523/JNEUROSCI.2615-05.2006. 16399703 PMC6674302

[acel70032-bib-0033] Krishnarajah, S. , F. Ingelfinger , E. Friebel , et al. 2022. “Single‐Cell Profiling of Immune System Alterations in Lymphoid, Barrier and Solid Tissues in Aged Mice.” Nature Aging 2, no. 1: 74–89. 10.1038/s43587-021-00148-x. 37118354

[acel70032-bib-0034] Li, C. , L. Zhu , J. X. Liu , et al. 2023. “Cordycepin Delays Postovulatory Aging of Oocytes Through Inhibition of Maternal mRNAs Degradation via DCP1A Polyadenylation Suppression.” Cellular and Molecular Life Sciences 80, no. 12: 372. 10.1007/s00018-023-05030-0. 38001238 PMC10674002

[acel70032-bib-0035] Liu, X. , Z. Liu , Z. Wu , et al. 2023. “Resurrection of Endogenous Retroviruses During Aging Reinforces Senescence.” Cell 186, no. 2: 287–304. 10.1016/j.cell.2022.12.017. 36610399

[acel70032-bib-0036] Lokka, E. , L. Lintukorpi , S. Cisneros‐Montalvo , et al. 2020. “Generation, Localization and Functions of Macrophages During the Development of Testis.” Nature Communications 11, no. 1: 4375. 10.1038/s41467-020-18206-0. PMC7463013 32873797

[acel70032-bib-0037] Lu, J. , R. Ahmad , T. Nguyen , et al. 2022. “Heterogeneity and Transcriptome Changes of Human CD8(+) T Cells Across Nine Decades of Life.” Nature Communications 13, no. 1: 5128. 10.1038/s41467-022-32869-x. PMC9436929 36050300

[acel70032-bib-0038] McQuattie‐Pimentel, A. C. , Z. Ren , N. Joshi , et al. 2021. “The Lung Microenvironment Shapes a Dysfunctional Response of Alveolar Macrophages in Aging.” Journal of Clinical Investigation 131, no. 4: 299. 10.1172/JCI140299. PMC7919859 33586677

[acel70032-bib-0039] Mogilenko, D. A. , I. Shchukina , and M. N. Artyomov . 2022. “Immune Ageing at Single‐Cell Resolution.” Nature Reviews Immunology 22, no. 8: 484–498. 10.1038/s41577-021-00646-4. PMC8609266 34815556

[acel70032-bib-0040] Munnur, D. , Q. Teo , D. Eggermont , et al. 2021. “Altered ISGylation Drives Aberrant Macrophage‐Dependent Immune Responses During SARS‐CoV‐2 Infection.” Nature Immunology 22, no. 11: 1416–1427. 10.1038/s41590-021-01035-8. 34663977

[acel70032-bib-0041] Natrajan, M. S. , A. G. de la Fuente , A. H. Crawford , et al. 2015. “Retinoid X Receptor Activation Reverses Age‐Related Deficiencies in Myelin Debris Phagocytosis and Remyelination.” Brain 138, no. 12: 3581–3597. 10.1093/brain/awv289. 26463675 PMC4668920

[acel70032-bib-0042] Nie, X. , S. K. Munyoki , M. Sukhwani , et al. 2022. “Single‐Cell Analysis of Human Testis Aging and Correlation With Elevated Body Mass Index.” Developmental Cell 57, no. 9: 1160–1176. 10.1016/j.devcel.2022.04.004. 35504286 PMC9090997

[acel70032-bib-0043] Oishi, Y. , and I. Manabe . 2016. “Macrophages in Age‐Related Chronic Inflammatory Diseases.” NPJ Aging and Mechanisms of Disease 2: 16018. 10.1038/npjamd.2016.18. 28721272 PMC5515003

[acel70032-bib-0044] Orozco, S. L. , S. P. Canny , and J. A. Hamerman . 2021. “Signals Governing Monocyte Differentiation During Inflammation.” Current Opinion in Immunology 73: 16–24. 10.1016/j.coi.2021.07.007. 34411882 PMC8648978

[acel70032-bib-0045] Palacio, L. , M. L. Goyer , D. Maggiorani , et al. 2019. “Restored Immune Cell Functions Upon Clearance of Senescence in the Irradiated Splenic Environment.” Aging Cell 18, no. 4: e12971. 10.1111/acel.12971. 31148373 PMC6612633

[acel70032-bib-0046] Qin, S. , X. Yao , W. Li , et al. 2023. “Novel Insight Into the Underlying Dysregulation Mechanisms of Immune Cell‐To‐Cell Communication by Analyzing Multitissue Single‐Cell Atlas of Two COVID‐19 Patients.” Cell Death & Disease 14, no. 4: 286. 10.1038/s41419-023-05814-z. 37087411 PMC10122452

[acel70032-bib-0047] Ren, X. , W. Wen , X. Fan , et al. 2021. “COVID‐19 Immune Features Revealed by a Large‐Scale Single‐Cell Transcriptome Atlas.” Cell 184, no. 7: 1895–1913. 10.1016/j.cell.2021.01.053. 33657410 PMC7857060

[acel70032-bib-0048] Rodier, F. , J. P. Coppe , C. K. Patil , et al. 2009. “Persistent DNA Damage Signalling Triggers Senescence‐Associated Inflammatory Cytokine Secretion.” Nature Cell Biology 11, no. 8: 973–979. 10.1038/ncb1909. 19597488 PMC2743561

[acel70032-bib-0049] Rodrigues, P. F. , A. Kouklas , G. Cvijetic , et al. 2023. “PDC‐Like Cells Are Pre‐DC2 and Require KLF4 to Control Homeostatic CD4 T Cells.” Science Immunology 8, no. 80: eadd4132. 10.1126/sciimmunol.add4132. 36827419 PMC10165717

[acel70032-bib-0050] Ross, J. B. , L. M. Myers , J. J. Noh , et al. 2024. “Depleting Myeloid‐Biased Haematopoietic Stem Cells Rejuvenates Aged Immunity.” Nature 628, no. 8006: 162–170. 10.1038/s41586-024-07238-x. 38538791 PMC11870232

[acel70032-bib-0051] Santiago, J. , J. V. Silva , M. G. Alves , P. F. Oliveira , and M. Fardilha . 2019. “Testicular Aging: An Overview of Ultrastructural, Cellular, and Molecular Alterations.” Journals of Gerontology Series A, Biological Sciences and Medical Sciences 74, no. 6: 860–871. 10.1093/gerona/gly082. 29688289

[acel70032-bib-0052] Scholler, N. , R. Perbost , F. L. Locke , et al. 2022. “Tumor Immune Contexture Is a Determinant of Anti‐CD19 CAR T Cell Efficacy in Large B Cell Lymphoma.” Nature Medicine 28, no. 9: 1872–1882. 10.1038/s41591-022-01916-x. PMC9499856 36038629

[acel70032-bib-0053] Sen, P. , A. Helmke , C. M. Liao , et al. 2020. “SerpinB2 Regulates Immune Response in Kidney Injury and Aging.” Journal of the American Society of Nephrology 31, no. 5: 983–995. 10.1681/ASN.2019101085. 32209589 PMC7217424

[acel70032-bib-0054] Shen, X. , C. Wang , X. Zhou , et al. 2024. “Nonlinear Dynamics of Multi‐Omics Profiles During Human Aging.” Nature Aging 4: 1619–1634. 10.1038/s43587-024-00692-2. 39143318 PMC11564093

[acel70032-bib-0055] Shi, C. , and E. G. Pamer . 2011. “Monocyte Recruitment During Infection and Inflammation.” Nature Reviews Immunology 11, no. 11: 762–774. 10.1038/nri3070. PMC3947780 21984070

[acel70032-bib-0056] Simnica, D. , N. Akyuz , S. Schliffke , et al. 2019. “T Cell Receptor Next‐Generation Sequencing Reveals Cancer‐Associated Repertoire Metrics and Reconstitution After Chemotherapy in Patients With Hematological and Solid Tumors.” Oncoimmunology 8, no. 11: e1644110. 10.1080/2162402X.2019.1644110. 31646093 PMC6791461

[acel70032-bib-0081] Stuart, T. , A. Butler , P. Hoffman , et al. 2019. “Comprehensive Integration of Single‐Cell Data.” Cell 177, no. 7: 1888–1902.e21. 10.1016/j.cell.2019.05.031. 31178118 PMC6687398

[acel70032-bib-0057] Suryadevara, V. , A. D. Hudgins , A. Rajesh , et al. 2024. “SenNet Recommendations for Detecting Senescent Cells in Different Tissues.” Nature Reviews Molecular Cell Biology 25, no. 12: 1001–1023. 10.1038/s41580-024-00738-8. 38831121 PMC11578798

[acel70032-bib-0058] Terekhova, M. , A. Swain , P. Bohacova , et al. 2023. “Single‐Cell Atlas of Healthy Human Blood Unveils Age‐Related Loss of NKG2C(+) GZMB(−) CD8(+) Memory T Cells and Accumulation of Type 2 Memory T Cells.” Immunity 56, no. 12: 2836–2854. 10.1016/j.immuni.2023.10.013. 37963457

[acel70032-bib-0059] Terrabuio, E. , E. Zenaro , and G. Constantin . 2023. “The Role of the CD8+ T Cell Compartment in Ageing and Neurodegenerative Disorders.” Frontiers in Immunology 14: 1233870. 10.3389/fimmu.2023.1233870. 37575227 PMC10416633

[acel70032-bib-0060] Wan, Y. , L. Piao , S. Xu , et al. 2023. “Cathepsin S Deficiency Improves Muscle Mass Loss and Dysfunction via the Modulation of Protein Metabolism in Mice Under Pathological Stress Conditions.” FASEB Journal 37, no. 8: e23086. 10.1096/fj.202300395RRR. 37428652

[acel70032-bib-0061] Wang, J. , I. Jelcic , L. Muhlenbruch , et al. 2020. “HLA‐DR15 Molecules Jointly Shape an Autoreactive T Cell Repertoire in Multiple Sclerosis.” Cell 183, no. 5: 1264–1281. 10.1016/j.cell.2020.09.054. 33091337 PMC7707104

[acel70032-bib-0062] Wang, L. , W. Hong , H. Zhu , et al. 2024. “Macrophage Senescence in Health and Diseases.” Acta Pharmaceutica Sinica B 14, no. 4: 1508–1524. 10.1016/j.apsb.2024.01.008. 38572110 PMC10985037

[acel70032-bib-0063] Wang, M. , Y. Yang , D. Cansever , et al. 2021. “Two Populations of Self‐Maintaining Monocyte‐Independent Macrophages Exist in Adult Epididymis and Testis.” Proceedings of the National Academy of Sciences of the United States of America 118, no. 1: 6117. 10.1073/pnas.2013686117. PMC7817195 33372158

[acel70032-bib-0083] Wang, Y. , R. Li , R. Tong , et al. 2025. “Integrating Single‐Cell RNA and T Cell/B Cell Receptor Sequencing with Mass Cytometry Reveals Dynamic Trajectories of Human Peripheral Immune Cells from Birth to Old Age.” Nature Immunology 26, no. 2: 308–322. 10.1038/s41590-024-02059-6. 39881000 PMC11785523

[acel70032-bib-0064] Wang, X. , Q. Liu , Z. Zhuang , et al. 2024. “Decoding the Pathogenesis of Spermatogenic Failure in Cryptorchidism Through Single‐Cell Transcriptomic Profiling.” Cell Reports Medicine 5, no. 9: 101709. 10.1016/j.xcrm.2024.101709. 39226895 PMC11528238

[acel70032-bib-0065] Wang, X. M. , J. Y. Zhang , X. Xing , et al. 2022. “Global Transcriptomic Characterization of T Cells in Individuals With Chronic HIV‐1 Infection.” Cell Discovery 8, no. 1: 29. 10.1038/s41421-021-00367-x. 35351857 PMC8964811

[acel70032-bib-0066] Winkler, I. , A. Tolkachov , F. Lammers , et al. 2024. “The Cycling and Aging Mouse Female Reproductive Tract at Single‐Cell Resolution.” Cell 187, no. 4: 981–998. 10.1016/j.cell.2024.01.021. 38325365

[acel70032-bib-0067] Wong, C. , and D. R. Goldstein . 2013. “Impact of Aging on Antigen Presentation Cell Function of Dendritic Cells.” Current Opinion in Immunology 25, no. 4: 535–541. 10.1016/j.coi.2013.05.016. 23806201 PMC3775944

[acel70032-bib-0080] Wu, T. , E. Hu , S. Xu , et al. 2021. “clusterProfiler 4.0: A Universal Enrichment Tool for Interpreting Omics Data.” Innovation 2, no. 3: 100141. 10.1016/j.xinn.2021.100141. 34557778 PMC8454663

[acel70032-bib-0068] Wu, M. , W. Tang , Y. Chen , et al. 2024. “Spatiotemporal Transcriptomic Changes of Human Ovarian Aging and the Regulatory Role of FOXP1.” Nature Aging 4, no. 4: 527–545. 10.1038/s43587-024-00607-1. 38594460 PMC11031396

[acel70032-bib-0069] Xia, K. , P. Luo , J. Yu , et al. 2024. “Single‐Cell RNA Sequencing Reveals Transcriptomic Landscape and Potential Targets for Human Testicular Ageing.” Human Reproduction 39, no. 10: 2189–2209. 10.1093/humrep/deae199. 39241251 PMC11447013

[acel70032-bib-0070] Yang, W. , L. B. Liu , F. L. Liu , et al. 2023. “Single‐Cell RNA Sequencing Reveals the Fragility of Male Spermatogenic Cells to Zika Virus‐Induced Complement Activation.” Nature Communications 14, no. 1: 2476. 10.1038/s41467-023-38223-z. PMC10148584 37120617

[acel70032-bib-0071] Yang, W. , C. Zhang , L. B. Liu , et al. 2024. “Immunocompetent Mouse Models Revealed That S100A4(+) Monocytes/Macrophages Facilitate Long‐Term Zika Virus Infection in the Testes.” Emerging Microbes & Infections 13, no. 1: 2300466. 10.1080/22221751.2023.2300466. 38164719 PMC10773650

[acel70032-bib-0072] Yousefzadeh, M. J. , R. R. Flores , Y. Zhu , et al. 2021. “An Aged Immune System Drives Senescence and Ageing of Solid Organs.” Nature 594, no. 7861: 100–105. 10.1038/s41586-021-03547-7. 33981041 PMC8684299

[acel70032-bib-0073] Zhan, Y. , Q. Huang , Z. Deng , et al. 2024. “DNA Hypomethylation‐Mediated Upregulation of GADD45B Facilitates Airway Inflammation and Epithelial Cell Senescence in COPD.” Journal of Advanced Research 68: 201–214. 10.1016/j.jare.2024.02.005. 38342401 PMC11785585

[acel70032-bib-0074] Zhang, W. , S. Xia , W. Xiao , et al. 2023. “A Single‐Cell Transcriptomic Landscape of Mouse Testicular Aging.” Journal of Advanced Research 53: 219–234. 10.1016/j.jare.2022.12.007. 36528294 PMC10658307

[acel70032-bib-0075] Zhao, S. , W. Zhu , S. Xue , and D. Han . 2014. “Testicular Defense Systems: Immune Privilege and Innate Immunity.” Cellular & Molecular Immunology 11, no. 5: 428–437. 10.1038/cmi.2014.38. 24954222 PMC4197207

[acel70032-bib-0076] Zheng, W. , S. Zhang , S. Jiang , et al. 2021. “Evaluation of Immune Status in Testis and Macrophage Polarization Associated With Testicular Damage in Patients With Nonobstructive Azoospermia.” American Journal of Reproductive Immunology 86, no. 5: e13481. 10.1111/aji.13481. 34192390

[acel70032-bib-0077] Zhou, C. , Q. Guo , J. Lin , et al. 2024. “Single‐Cell Atlas of Human Ovaries Reveals the Role of the Pyroptotic Macrophage in Ovarian Aging.” Advanced Sciences 11, no. 4: e2305175. 10.1002/advs.202305175. PMC10811476 38036420

[acel70032-bib-0078] Zhu, H. , J. Chen , K. Liu , et al. 2023. “Human PBMC scRNA‐Seq‐Based Aging Clocks Reveal Ribosome to Inflammation Balance as a Single‐Cell Aging Hallmark and Super Longevity.” Science Advances 9, no. 26: eabq7599. 10.1126/sciadv.abq7599. 37379396 PMC10306289

[acel70032-bib-0079] Zhuang, J. , X. Li , J. Yao , et al. 2023. “Single‐Cell RNA Sequencing Reveals the Local Cell Landscape in Mouse Epididymal Initial Segment During Aging.” Immunity & Ageing 20, no. 1: 21. 10.1186/s12979-023-00345-9. 37170325 PMC10173474

