# Supplementary figures and images for: A Single‐Cell Transcriptome Atlas Characterizes the Immune Landscape of Human Testes During Aging
Source: Aging Cell. 2025 Mar 6;24(6):e70032. doi: 10.1111/acel.70032 (PMC12151895; doi:10.1111/acel.70032)

Figure S1. Single-cell analysis of immune cells in human testis during aging.

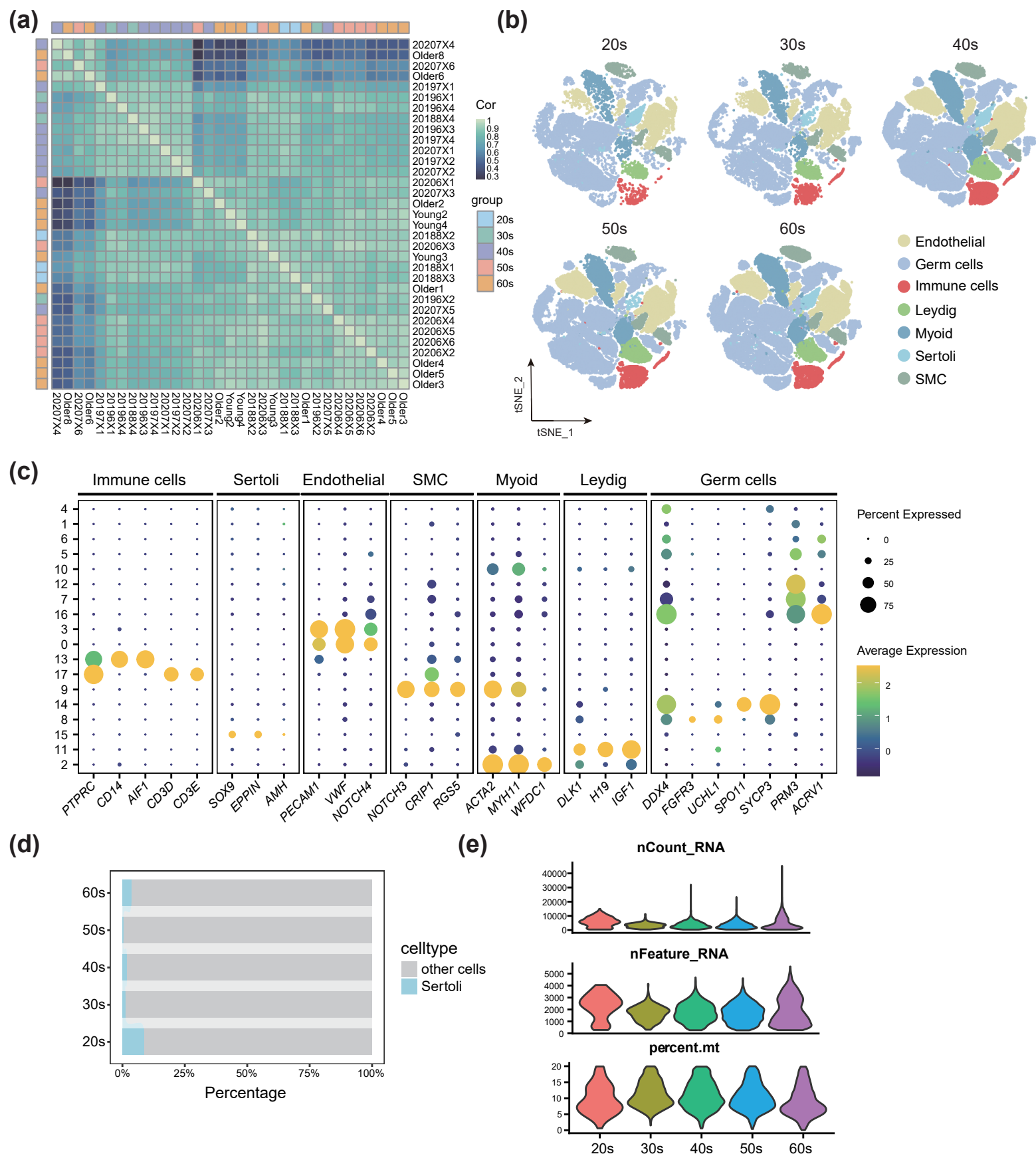

Supplement: Supplementary file 1 — Figure S1Single‐cell analysis of immune cells in human testis during aging. (a) Heatmap showing the correlation between samples. The color scale represents the correlation. (b) Separate t‐SNE plot of major testicular cell types in groups. SMC, smooth muscle cells. (c) Expression of selected markers identifying major testicular cell types. Expression level as shown on the color at the right. (d) Bar plot showing the percentage of Sertoli cells in each group. (e) Violin plots showing the UMIs/genes/percent.mito in overall cells from left to right. [file ACEL-24-e70032-s001.pdf]

Figure S2. Aging-related features increased in Group B.

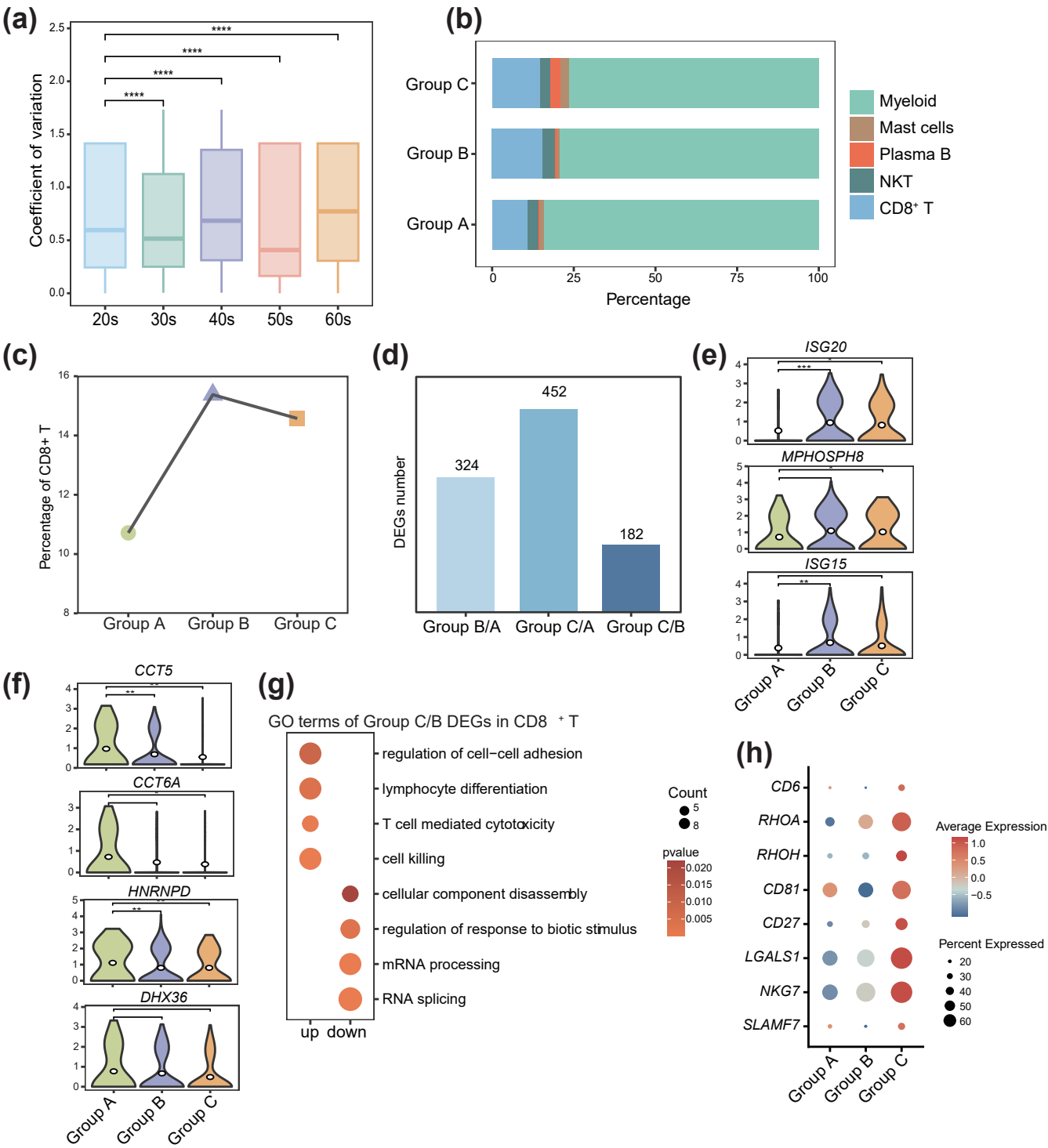

Supplement: Supplementary file 2 — Figure S2Age‐related features increased in Group B. (a) Bar plot showing the value of coefficient of variation (cv) of immune cells. Each group randomly selected 3 samples (n = 3). Box indicates range from 25th to 75th percentile; center lines indicate medium. The p value was calculated by Student’s t‐test. ****p < 0.0001. (b) Bar plot showing the percentage of immune subtypes in three age groups. (c) Line plot showing the percentage of CD8+ T cells in immune cells among three age groups. Color indicates each age group. (d) The DEGs number of CD8+ T cells in Group B/A, C/A and C/B. (e) Violin plots showing the expression of genes in negative regulation of viral genome replication from (Figure 2g). (f) Violin plots showing the expression of genes in telomere maintenance from (Figure 2g). (g) Representative GO terms enrichment of DEGs in Group C/B from (d) and their associated p value. (h) Dot plots showing the selected genes of pathways from (g). [file ACEL-24-e70032-s004.pdf]

Figure S3. MRC1<sup>hi</sup> TRM remain relatively stable, with a decline of function during aging.

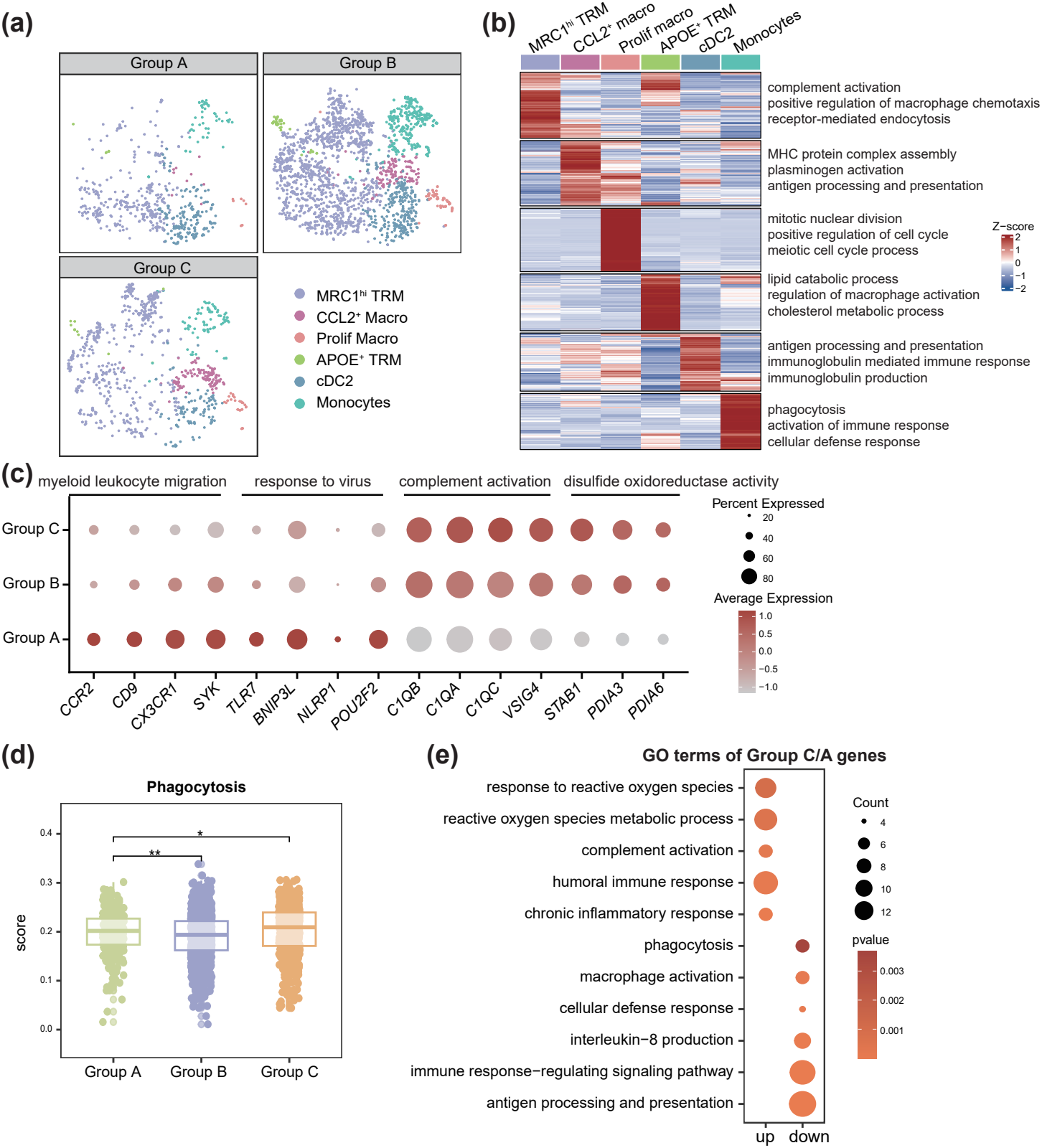

Supplement: Supplementary file 3 — Figure S3MRC1hi TRM remain relatively stable, with a decline of function during aging. (a) Separate t‐SNE plot of myeloid subtypes in each age group. (b) Left: heatmap showing the top 100 differentially expressed genes of each cell cluster from (Figure 3a). The scaled gene expression levels are colored according to Z score. Right: the corresponding top two GO terms enriched in the marker genes of each cell cluster with Z score colored according to the color key at the right. (c) Dot plot showing the expression of genes in the pathways from (Figure 3f) in three age groups. The enriched pathway on the top. Color represents the average expression. (d) Box plot showing the signature score of phagocytosis of monocytes among three age groups. Color indicates the age groups, box indicates range from 25th to 75th percentile; center lines indicate medium, dot represent individual cells. The p value was calculated by Student’s t‐test. **p ≤ 0.01, *p ≤ 0.05. (e) Dot plots showing the GO terms of DEGs of Group A to Group B in MRC1hi TRM. [file ACEL-24-e70032-s002.pdf]

Figure S4 Monocytes accumulated and contributed low-grade inflammation during aging.

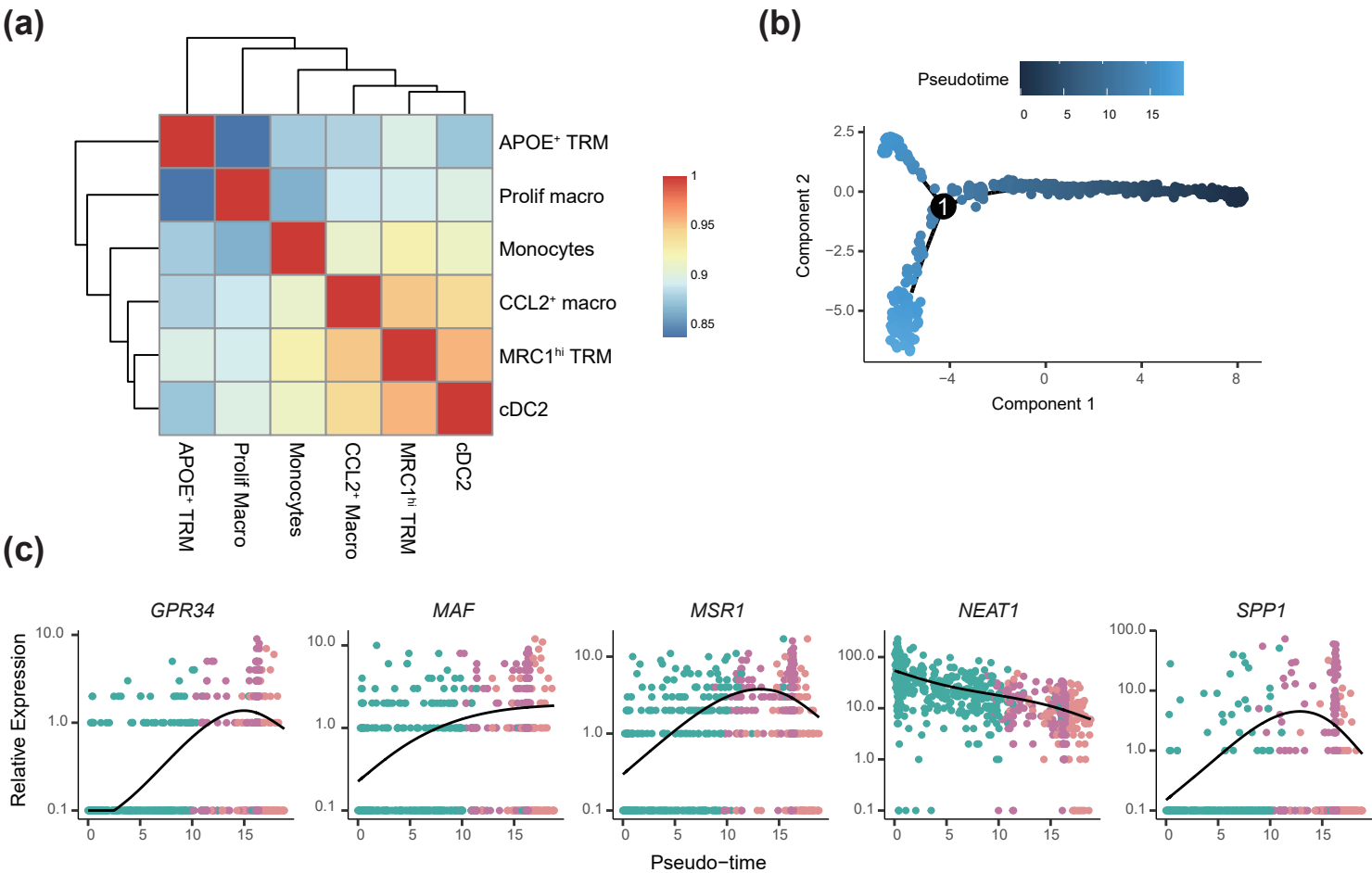

Supplement: Supplementary file 4 — Figure S4Monocytes accumulated and contributed low‐grade inflammation during aging. (a) Heatmap showing the correlations of each myeloid subtypes. (b) CCL2+ macrophages, Prolif macrophages and monocytes pseudotime analysis using Monocle2. (c) The dynamic expression of GPR34, MAF, MSR1, NEAT1, SPP1 along the pseudotime trajectory. [file ACEL-24-e70032-s005.pdf]

Figure S5. Age-related changes in cDC2 cells.

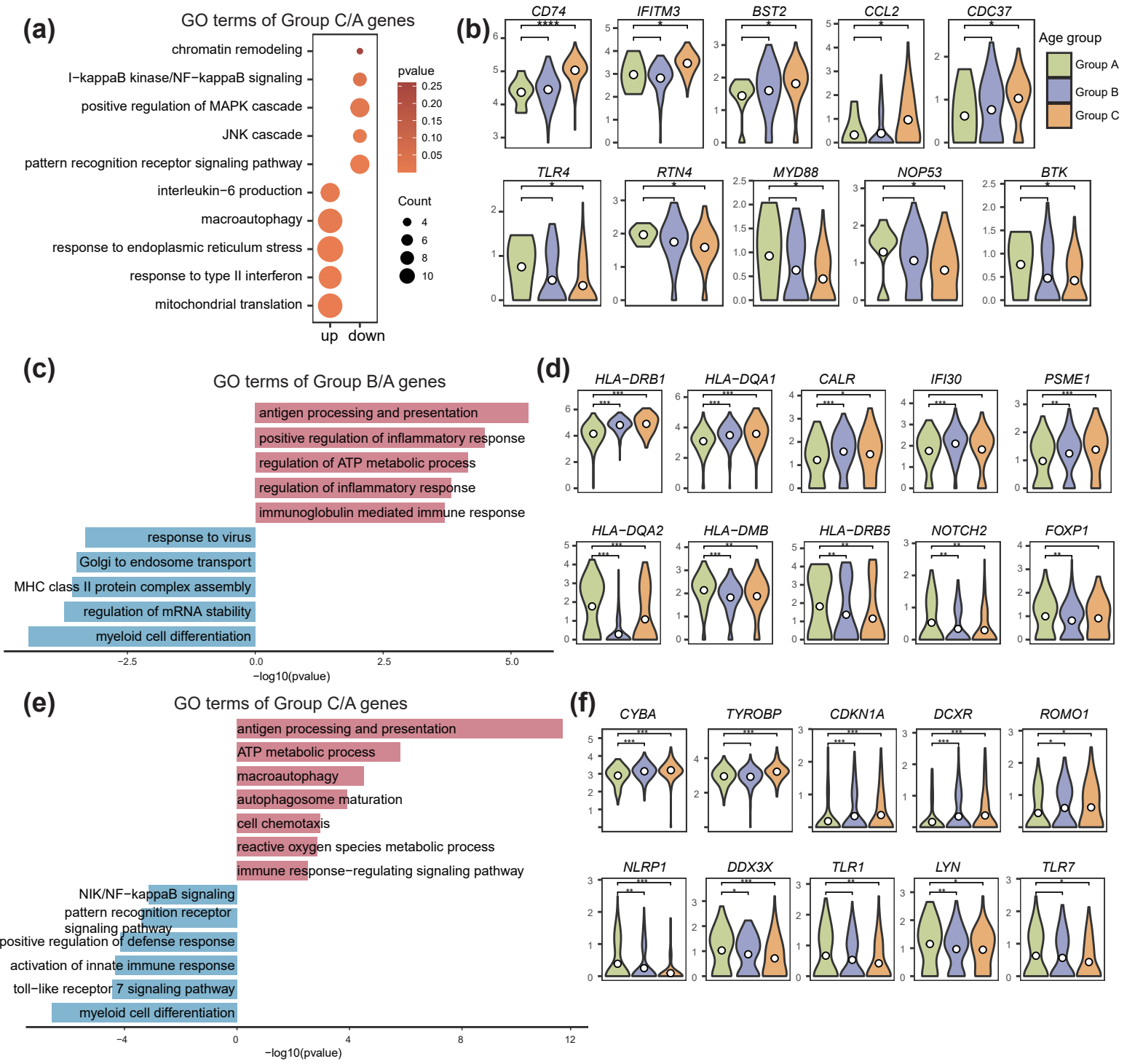

Supplement: Supplementary file 5 — Figure S5 Age‐related changes in cDC2 cells. (a) Representative GO terms enrichment of 304 unique DGEs of Group C/A from (Figure 5f) and their associated p value. (b) Violin plots showing the expression level of selected genes from (a) in CCL2+ macrophages. The white dot inside the violin represents the mean. Wilcox.test, ****p ≤ 0.0001; *p ≤ 0.05; ns, p > 0.05. (c) Representative GO terms enrichment of DEGs in Group B/A in cDC2 cells and their associated p value. (d) Violin plots showing the expression level of selected genes from (c) The white dot inside the violin represents the mean. Wilcox.test, ; ***p ≤ 0.001; **p ≤ 0.01; *p ≤ 0.05; ns, p > 0.05. (e) Representative GO terms enrichment of DEGs in Group C/A in cDC2 cells and their associated p value. (f) Violin plots showing the expression level of selected genes from (e). The white dot inside the violin represents the mean. Wilcox.test, ; ***p ≤ 0.001; **p ≤ 0.01; *p ≤ 0.05; ns, p > 0.05. [file ACEL-24-e70032-s003.pdf]
